# A Review of Optical Interferometry for High-Precision Length Measurement

**DOI:** 10.3390/mi16010006

**Published:** 2024-12-24

**Authors:** Guangyao Huang, Can Cui, Xiaoyang Lei, Qixue Li, Shuhua Yan, Xinghui Li, Guochao Wang

**Affiliations:** 1 College of Intelligence Science and Technology, National University of Defense Technology, Changsha 410073, China; huang-gy22@mails.tsinghua.edu.cn (G.H.); leixiaoyang@nudt.edu.cn (X.L.); liqixue12@outlook.com (Q.L.); yanshuhua996@163.com (S.Y.); 2 Tsinghua Shenzhen International Graduate School, Tsinghua University, Shenzhen 518055, China; cuic23@mails.tsinghua.edu.cn (C.C.); li.xinghui@sz.tsinghua.edu.cn (X.L.)

**Keywords:** laser interferometry, grating interferometry, optical frequency comb, length measurement

## Abstract

Optical interferometry has emerged as a cornerstone technology for high-precision length measurement, offering unparalleled accuracy in various scientific and industrial applications. This review provides a comprehensive overview of the latest advancements in optical interferometry, with a focus on grating and laser interferometries. For grating interferometry, systems configurations ranging from single-degree- to multi-degree-of-freedom are introduced. For laser interferometry, different measurement methods are presented and compared according to their respective characteristics, including homodyne, heterodyne, white light interferometry, etc. With the rise of the optical frequency comb, its unique spectral properties have greatly expanded the length measurement capabilities of laser interferometry, achieving an unprecedented leap in both measurement range and accuracy. With regard to discussion on enhancement of measurement precision, special attention is given to periodic nonlinear errors and phase demodulation methods. This review offers insights into current challenges and potential future directions for improving interferometric measurement systems, and also emphasizes the role of innovative technologies in advancing precision metrology technology.

## 1. Introduction

Length is one of the seven fundamental physical quantities, and high-precision length measurement is a crucial foundation for modern industry and scientific research [1,2,3,4]. From the manufacturing of large-scale equipment to the fabrication of nanochips, and from the validation of physical theories to the definition of fundamental constants, length measurement plays an indispensable role. Among the various techniques for length measurement, optical methods are the most fundamental approaches. Moreover, the meter is defined in terms of the speed of light in a vacuum. In 1893, the 17th General Conference on Weights and Measures defined the meter as the distance light travels in a vacuum in 1/299,792,458 s, establishing a traceable relationship between the meter and the second based on the speed of light [5,6]. This further illustrates the fundamental importance of using optical methods as a means of length measurement.

The main optical length measurement methods include pulsed Time-Of-Flight (TOF) and optical interferometry. Pulsed TOF measures the time it takes for a light pulse to travel to a target and return to the detector, from which the target distance can be calculated [7,8,9,10]. Pulsed TOF can directly measure absolute distances, with ranges extending beyond kilometers, but its resolution is typically limited to the millimeter scale due to the bandwidth constraints of photodetectors. Optical interferometry, on the other hand, utilizes optical coherence to derive distance information. With the characteristics of high precision, high resolution and traceability, optical interferometry has found widespread applications in micron- and nanometer-level accuracy measurements.

Interferometric methods can be broadly categorized into two main approaches: grating interferometry and laser interferometry. Each of these methods has its distinct operational principles and application focus. Grating interferometry involves the use of diffraction gratings to create interference patterns [11,12,13]. This approach excels in achieving even higher precision and allows for multi-dimensional measurement expansion. Grating interferometries can perform measurements with sub-nanometer accuracy [14], making them suitable for tasks where ultra-high precision is critical. Moreover, their adaptability in multi-axis and multi-degree-of-freedom measurements makes them a preferred choice for applications requiring complex spatial displacement tracking, such as in advanced manufacturing and optical alignment systems [15]. On the other hand, laser interferometry uses laser wavelength as a reference, which can be traced back to the “meter” standard. This method is particularly well suited for long-range displacement measurements, benefiting from its high resolution and precision over extended distances. One of its notable advantages is the ability to operate efficiently in vacuum environments, making it an ideal choice for applications that require precise measurements over long travel ranges, such as metrology in semiconductor manufacturing, aerospace component testing, and gravitational wave detecting. The emergence of the new laser source optical frequency comb has injected powerful vitality into the development of laser interferometry. Since the optical frequency comb was first applied in absolute length measurement in 2000, 24 years of development have greatly enriched the means of precision length measurement. The excellent time-frequency domain characteristics of the optical frequency comb, such as wide spectral range, narrow pulse width, and high repetition frequency stability, provide a new technical means for absolute length measurement. The optical frequency comb can be used directly as a measuring light source for length measurement, or as a precision optical frequency ruler to calibrate laser frequency [16,17].

Both grating and laser interferometries have been successfully applied in various high-precision fields. In the domain of photolithography, they are essential for ensuring the accurate positioning of stages [18], which directly affects the resolution and accuracy of semiconductor patterning [19]. Similarly, in high-precision machining, interferometries are used for real-time feedback control to maintain extremely tight tolerances, enhancing product quality and consistency [20,21,22]. Additionally, they play a critical role in surface profile measurements, where the precise and non-contact evaluation of surface features is required. Beyond manufacturing, interferometric techniques are also crucial in the development and inspection of integrated circuits [23], as they ensure the integrity and precision of circuit patterns during fabrication. Furthermore, the aerospace industry leverages interferometry for tasks ranging from the calibration of precision components to the monitoring of structural deformations.

This paper systematically reviews the existing optical interferometric length measurement methods, classifies them based on their measurement principles, and summarizes their current research status and development trends. Furthermore, it compares the advantages and disadvantages of different methods, analyzes the key factors affecting interferometric length measurement, and discusses potential future research directions and development prospects.

## 2. Grating Interferometry

### 2.1. One-Degree-of-Freedom Grating Interferometry

**Figure 1:**
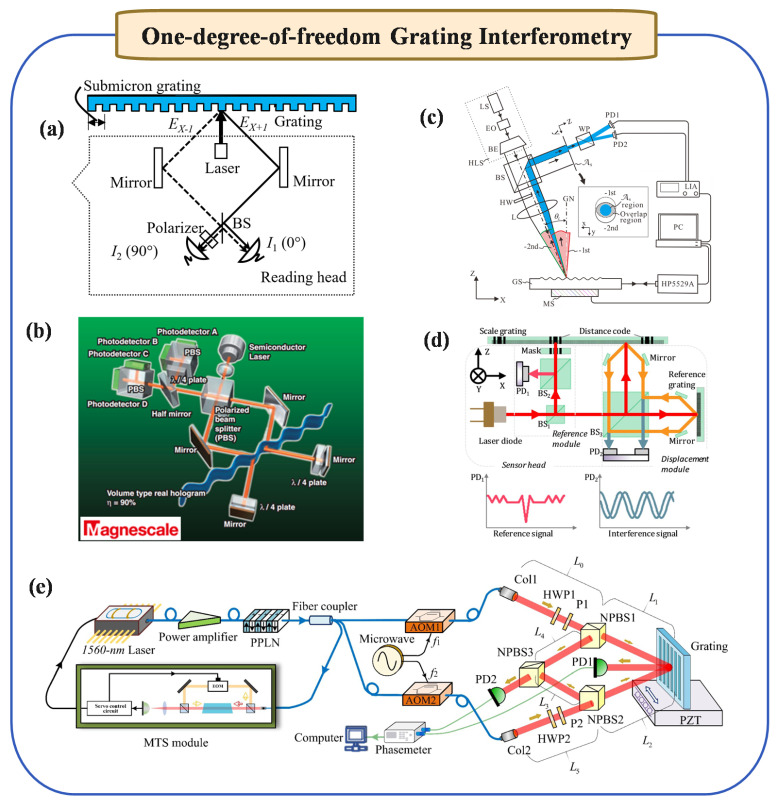
Schematic diagram of one-degree-of-freedom grating interferometry. (**a**) Basic measurement principle of grating interferometry [24]. (**b**) Magnescale’s Grating Interferometry Products [25]. (**c**) Littrow grating interferometry in 2013, reprinted from [26]. (**d**) Absolute 2D optical encoders in 2019, reprinted from [27]. (**e**) Quasi-common-path heterodyne grating interferometry in 2024, reprinted with permission from [14], copyright 2024 IEEE.

The development of grating interferometry originated from one-dimensional diffraction gratings. The basic principle involves combining the positive and negative first-order diffracted light to produce interference. Specifically, as illustrated in Figure 1a, the grating interferometer operates by introducing a relative phase shift between two diffracted beams. When the grating moves, this motion induces a change in the relative phase of the observed signals ($I_{1}$ and $I_{2}$). By measuring this phase shift, the displacement of the grating can be quantitatively determined. The displacement Δx can be extracted from the measured phase shift Δφ using the following equation:

(1) $$\Delta x=\frac{g\Delta \varphi }{2\pi N}$$

where *N* is the optical subdivision, *g* is the grating period, and Δφ represents the phase shift induced by the grating’s movement. Thus, by monitoring the interference signals and determining the corresponding phase shift, precise displacement measurements can be achieved [24].

Later, some scholars proposed some new solutions to improve the performance of single-degree-of-freedom grating interferometries as follows: 1. Reduced Grating Period and Enhanced Optical Subdivision: As shown in Figure 1b, by employing a smaller grating period and higher optical subdivision, the signal period is minimized, enabling resolutions down to 0.017 nm [25]. This approach allows for finer resolution in displacement measurements. 2. Self-Collimating Incidence Structure: The structural adaptation of a self-collimating incidence method renders the system less sensitive to movements along the grating’s normal direction, thereby enhancing measurement stability [28]. This structural modification is crucial for improving the overall reliability of the measurements. 3. New Optical Path Structure Design: Hsieh et al. [29] proposed a novel heterodyne grating interferometry with quasi-common-optical-path design which achieves sub-3 nm resolution and high system stability, and addresses key error effects.

In recent years, many scholars have proposed some new high-performance one-degree-of-freedom measurement methods. As shown in Figure 1c, in order to improve the measurement range in the z-direction, Wu et al. [26] proposed the Littrow grating interferometry. In response to the error problem in grating interferometries, in 2015, Lin et al. [30] proposed a symmetrical short grating period heterodyne grating interferometry with a final resolution better than 2 nm. Later in 2017, Xing et al. [31] proposed a spatially separated heterodyne grating interferometry, reducing the periodic nonlinear error to 0.086 nm. Recently, as shown in Figure 1e, Wang et al. [14] proposed a quasi-common optical path and frequency-stabilized heterodyne grating interferometry that can meet the accuracy of sub-nanometer measurement. Li et al. [32] introduced a two-probe optical encoder designed for the absolute positioning of precision stages, utilizing an improved scale grating. The encoder enhances positioning accuracy by effectively mitigating common-mode errors, proving valuable for high-precision motion control applications.

For absolute measurement needs, as shown in Figure 1d, Shi et al. [27] adopted a hybrid positioning method to integrate absolute coding information into the grating, achieving high-precision absolute measurement. Then, Shi et al. [33] proposed a linear encoder for absolute precision-positioning, by integrating a single-track grating scale with a two-probe reading head. This encoder can accurately measures displacement and position, achieving over 0.22 µm positioning accuracy and 1 µm incremental displacement precision, validated against a laser interferometry reference.

Despite the sub-nanometer precision achievable by single-degree grating interferometry, it is primarily suited for use in stacked stage setups and is not applicable in scenarios requiring multi-degree positioning, such as in lithography machines. Therefore, some scholars began to study multi-degree-of-freedom ultra-precision positioning grating interferometries, which will be introduced in detail below.

### 2.2. Two-Degree-of-Freedom Grating Interferometry

**Figure 2:**
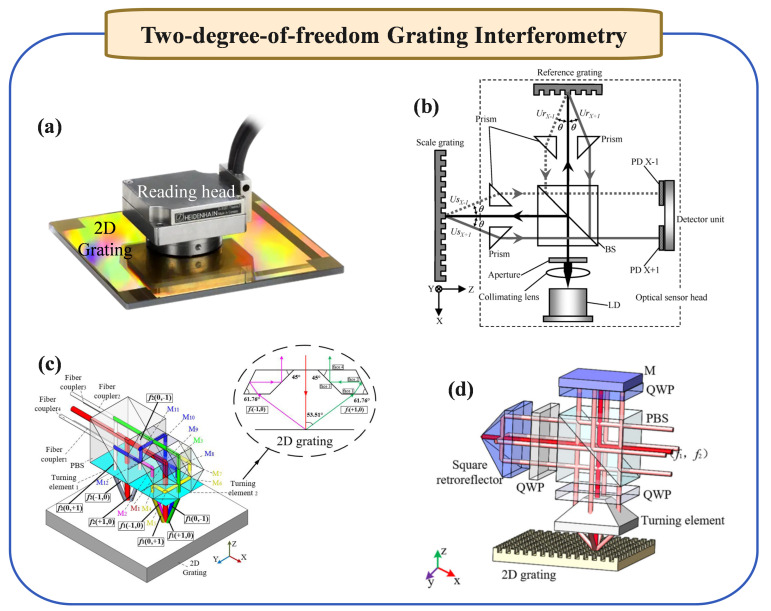
Schematic diagram of two-degree-of-freedom grating interferometry. (**a**) Heidenhain’s 2D grating interferometry PP281 [35]. (**b**) 2D grating interferometry for in-plane and out-of-plane measurements, reprinted with permission from [36], copyright 2010 Elsevier. (**c**) Double-spatial heterodyne 2D grating interferometry in 2022, reprinted with permission from [37], copyright 2022 Elsevier. (**d**) Single-incident beam 2D measurement systems in 2021, reprinted from [38].

To address the challenge of multi-degree measurement, the development of two-degree grating interferometry capable of simultaneously measuring movements in two displacement degrees has been pivotal. As proposed by Xia et al. [34], this interferometry uses two-dimensional diffraction gratings with consistent groove patterns in orthogonal directions. Based on the principle of single-frequency laser homodyne interferometry, it obtains phase changes due to Doppler frequency shifts in both diffraction directions, thereby calculating displacements in two directions. In addition, some internationally renowned companies, such as Heidenhain, have also developed high-precision two-dimensional grating interferometers and achieved excellent market success, as shown in Figure 2a.

To address the challenge of concurrent measurements in precision linear stages, as shown in Figure 2b, Kimura et al. [36] developed a two-degree-of-freedom (two-DOF) linear encoder. This innovative system is adept at simultaneously measuring position along the X-axis and straightness along the Z-axis. Constructed with a reflective-type scale grating and an optical sensor head, this encoder, through interference signals generated by diffracted beams, achieves sub-nanometer resolution in both axes. Furthermore, to enhance the quality of interference signals and resistance to environmental fluctuations, heterodyne interferometry-based two-degree grating scales have been proposed. Hsu et al. [39] introduced a new method for one-dimensional and two-dimensional in-plane displacement measurement based on heterodyne grating interferometry. This innovative optical setup minimizes airstream disturbances and environmental vibrations, achieving high stability and low measurement error. The method’s sensitivity reaches the sub-picometer level, and with a controlled isolation system, it attains a resolution of about 0.5 nm within a 250 µm displacement range, enabling simultaneous 2D in-plane measurements with a single interferometry. Wang et al. [40] proposed a cutting-edge heterodyne grating interferometry system capable of simultaneously measuring long strokes (hundreds of millimeters) in-plane and short strokes (hundreds of micrometers) out-of-plane. This system achieves a displacement resolution of 1.63 nm in the x-direction and 0.75 nm in the z-direction, with primary accuracy tests indicating a standard deviation of 6.37 nm in-plane and 3.69 nm out-of-plane. Lin et al. [41] explored a gold-coated cross-grating to enhance diffraction efficiency, signal contrast, and optical subdivision in heterodyne grating interferometries (HGIs). Through diffraction and polarization analyses, it achieves up to 18.32% efficiency with 100% signal contrast theoretically, offering an eightfold optical subdivision applicable to 2D configurations, validated by experimental consistency. Hsieh et al. [42] proposed a grating-based interferometry for 6-DOF displacement and angle measurements, using heterodyne, grating shearing, and Michelson techniques for enhanced resolution, achieving about 2 nm in displacement and 0.05 µrad in angular measurements. Zhu et al. [43] introduced dual-comb ranging, a technique leveraging phase resolution and frequency accuracy for high-precision, fast-rate distance measurement, overcoming conventional tool limitations, with promising diverse applications. Yang et al. [44] proposed a two-degree-of-freedom fiber-coupled heterodyne grating interferometry, designed for wafer stage displacement measurement in lithography machines. It features a large rotation range and uses fibers without couplers for high-contrast signal reception. The system, validated experimentally and through ZEMAX ray tracing simulations, includes a reference beam to suppress thermal drift, achieving sub-nanometer displacement stability of 0.246 nm and 0.465 nm in-plane and out-of-plane, respectively, within 30 s. As shown in Figure 2c,d, Yin et al. [37,38] present a high-precision 2D grating displacement system using double-spatial heterodyne optical path interleaving, achieving a measurement resolution within 3 nm and enhanced accuracy suitable for engineering testing.

Two-degree planar grating interferometries play a significant role in addressing issues such as Abbe errors and reducing the size of measurement systems. However, due to their inability to measure out-of-plane degrees of freedom (Z-axis), they still cannot meet the six-degree positioning requirements of current lithography machine wafer stages. Nevertheless, it is noteworthy that these developments provide valuable insights for the research and development of multi-degree grating interferometry.

### 2.3. Three-Degree-of-Freedom Grating Interferometry

**Figure 3:**
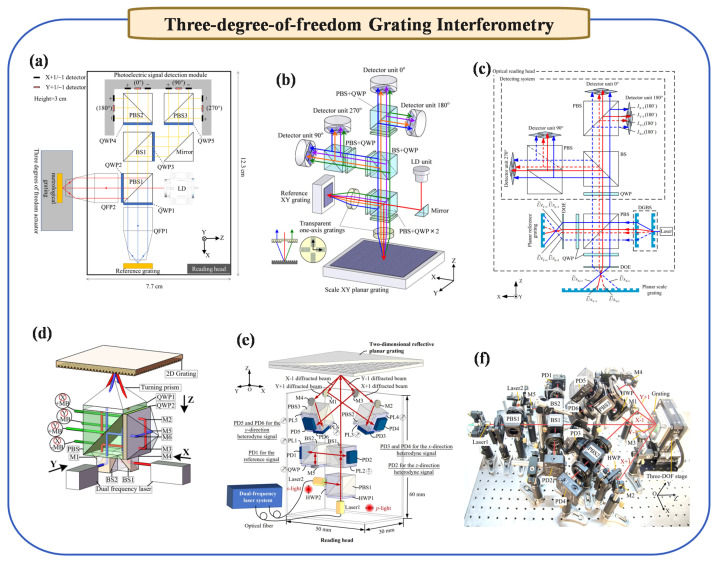
Schematic diagram of three-DOF grating interferometry. (**a**) Compact, high-precision 3D optical encoders in 2023, reprinted from [45]. (**b**) Three-degree-of-freedom optical encoder in 2012, reprinted with permission from [46], copyright 2012 Elsevier. (**c**) High-resolution, long-range, three-degree-of-freedom grating encoder in 2017, reprinted from [47]. (**d**) Littrow equipath grating interferometry in 2022, reprinted from [48]. (**e**,**f**) Reflective sub-nanometer-precision heterodyne grating interferometry in 2022, reprinted with permission from [49], copyright 2022 IEEE.

Based on the two-dimensional plane grating systems discussed earlier, which effectively overcome Abbe errors and facilitate multi-degree measurements with compact structures, advancements have been made to develop three- and six-degree measurement systems. These systems, capable of measuring in-plane displacements (XY) and out-of-plane displacements (Z), as well as three angular displacements, are well suited to meet the high-precision, multi-degree measurement requirements of photolithography machines. Next, we first introduce the progress of three-degree-of-freedom positioning methods, as shown in Figure 3.

Homodyne grating interferometry operates on the principle of single-frequency laser interference. The generated signals are DC, and displacements are calculated from the periodic changes in signal amplitude. First, Gao et al. [50] proposed a new type of homodyne three-degree-of-freedom interferometry. Replacing conventional Michelson interferometry plane mirrors, a three-axis displacement sensor using two sinusoidal XY-grid mirrors with identical pitches and amplitudes generates interference signals for X-, Y-, and Z-axis displacement measurements. This approach, confirmed experimentally, achieves nanometric resolution in all three axes. Subsequently, as shown in Figure 3b, Kimura et al. [46] presented a three-axis surface encoder with sub-nanometric resolutions for stage motion measurement, utilizing XY planar gratings and an optical sensor head, achieving enhanced resolution in X-, Y-, and Z-directions. Then, Shimizu et al. [51] proposed a four-probe three-degree-of-freedom grating interferometry and conducted application tests on large-area spliced gratings. However, it is noteworthy that Z-axis displacement measurement in this system is somewhat limited, as large Z displacements can cause the light spot on the detector to move, weakening the measurement signal. Subsequently, as shown in Figure 3c, Lin et al. [47] proposed a wide-range three-axis grating encoder with nanometric resolution, capable of measuring X-, Y-, and Z-axial motions of a stage, using a reflective-type planar scale grating and an innovative optical design, achieving a Z-axial displacement resolution of 4 nm. Recently, as shown in Figure 3a, Wang et al. [45] proposed a compact, high-precision 3-DOF grating encoder, based on quadrangular frustum pyramid prisms, to address space constraints in displacement measurements. Achieving simultaneous X, Y, and Z measurements within specified ranges, it demonstrates an average accuracy below 500 nm, optimizing multi-DOF encoder applications in precision contexts. Recently, as shown in Figure 3d, Yin et al. [48] used the Littrow optical path structure to realize the interference optical path.

Currently, there are two main technological approaches to heterodyne three-degree grating interferometry. The first solution involves adding an axis to the planar XY two-degree heterodyne grating interferometry. Hsieh et al. [52] proposed a heterodyne grating-based interferometry for 3-DOF displacement measurement, combining the advantages of heterodyne and grating interferometry, capable of nanometric resolution and millimetric range in sensing 3-DOF stage displacement, demonstrated through experimental results. Another solution is to use a dual-frequency laser measurement system, of which there are two representative works. Lin et al. [53] proposed a three-dimensional grating displacement measurement system using a dual-frequency laser. Then, Tan et al. [54] presented a three-dimensional displacement measurement device using a dual-frequency laser and diffraction grating. This methods fully expands the displacement range of the Z-axis and improves the anti-interference ability of the measurement signal. Recently, as shown in Figure 3e,f, Zhu et al. [49] used a reflective optical structure to achieve sub-nanometer precision three-degree-of-freedom measurement, which is expected to be applied in fields such as lithography.

### 2.4. Multi-Degree-of-Freedom Grating Interferometry

In the realm of precision measurement, accomplishing multi-degree measurements with high precision is universally acknowledged as a formidable task. Systems based on homodyne and heterodyne interferometry, designed for this purpose, often exhibit complex structure. Typically, these systems are differentiated into two types: those that utilize a single optical head and others that employ multiple optical heads for six-degree measurements. The following are detailed introductions.

#### 2.4.1. Multi-DOF Grating Interferometry (Single Optical Head)

For the single-optical-head approach, the system fundamentally builds upon a standard linear displacement measurement setup and then integrates an additional angular sensing component. In other words, starting from the basic configuration used to measure linear motion, an angle measurement module is introduced into the same optical path, allowing both displacement and small angle changes to be detected simultaneously.

**Figure 4:**
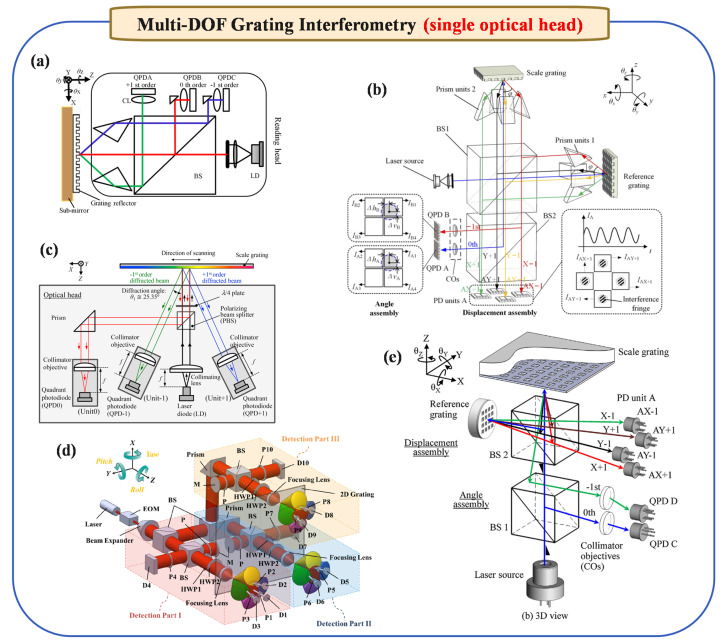
Principle diagram of multi-DOF grating interferometry (single optical head). (**a**) Absolute high-precision and multi-DOF optical encoders in 2022, reprinted from [59]. (**b**) Two-channel six-degree-of-freedom heterodyne grating interferometry in 2021, reprinted from [58,61]. (**c**) Multidimensional grating interferometry for evaluating grating period error in 2021, reprinted with permission from [57], copyright 2021 Elsevier. (**d**) Grating interferometry of six-DOF in 2015, reprinted from [42]. (**e**) Six-axis optical encoder in 2013, reprinted with permission from [56], copyright 2013 Elsevier.

By expanding the three-degree-of-freedom measurement method, Gao et al. [55] introduced a novel autocollimator capable of detecting three-axis angular error motions (pitch, yaw, and roll) in precision stages, using a laser diode and grating reflector, and achieving 0.01 arc-second resolution in angular motion detection across all axes. Systems that evolved thereafter are extensively visualized and summarized in Figure 4, which outlines several representative single-optical-head grating interferometry setups applied to multi-DOF measurements. As one such advancement, Li et al. [56] developed a multi-axis surface encoder (see Figure 4e) capable of measuring six-DOF translational and angular motions. Using a 405 nm blue laser diode, it features a compact sensor head and a planar scale grating for enhanced motion detection. This configuration reduces Abbe errors and mitigates environmental impacts, achieving linearity over 80 µm and 5 nm stability within 300 s. This performance makes it ideal for multi-axis ultra-precision positioning in nanoscience and technology applications.

Subsequent innovations have focused on enhancing both resolution and measurement dimensions. For instance, Figure 4d depicts a six-DOF grating interferometry configuration reported in 2015 [42], while Figure 4c showcases a multidimensional grating interferometry system proposed in 2021 [57] for evaluating grating period errors. Likewise, Figure 4b introduces a two-channel six-degree-of-freedom heterodyne grating interferometry from 2021 [58], demonstrating the feasibility of simultaneous multi-DOF sensing within a single optical head. More recently, Wang et al. [59,60] have advanced this concept further, as illustrated in Figure 4a, with a four-DOF absolute grating encoder capable of measuring three-axis pose (θx, θy, θz) and Z-axis position. By employing a stationary reading head and a movable grating reflector, and by analyzing diffracted beams via quadrant photodetectors, this encoder achieves sub-arcsecond and sub-micrometer accuracy.

#### 2.4.2. Multi-DOF Grating Interferometry (Multi-Optical Heads)

In contrast, the multi-optical-head method relies on several distinct linear displacement measurement modules placed in carefully arranged positions. By analyzing the relative shifts recorded by each of these modules, both linear and angular changes can be deduced. In other words, multiple measurement beams—each independently capturing linear displacement—are combined mathematically to extract both straight-line motion and rotational movement about various axes.

**Figure 5:**
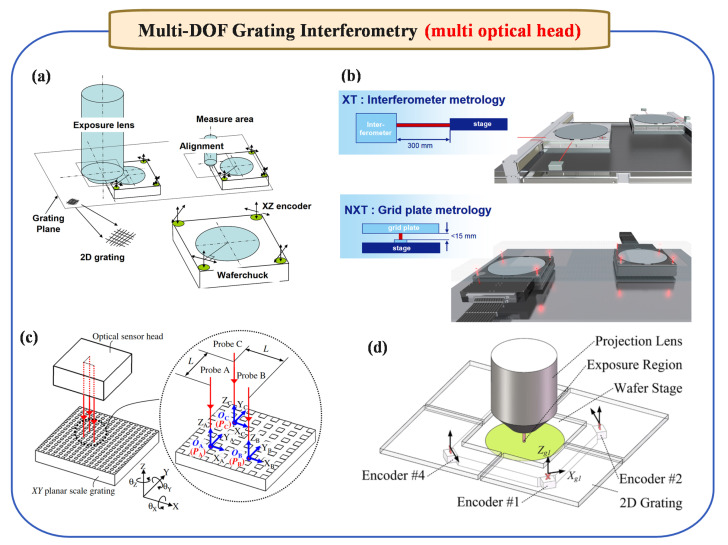
Principle diagram of multi-DOF grating interferometry (multi-optical head). (**a**) ASML’s multi-optical head solution in 2009, reprinted from [62]. (**b**) Comparison of optical path lengths between laser interferometry and grating interferometry in 2010, reprinted from [63]. (**c**) Right-angle multi-optical head layout scheme in 2014, reprinted from [64]. (**d**) Multi-optical head arrangement scheme for the wafer stage of lithography in 2019, reprinted with permission from [65], copyright 2019 Elsevier.

From early explorations to more recent advancements, multi-optical-head solutions have continually evolved to enhance multi-DOF measurement capabilities (see Figure 5). For instance, ASML’s multi-optical-head solution in 2009 [62] (Figure 5a) established a foundation for integrating multiple optical paths, while comparative studies on optical path lengths between laser and grating interferometry in 2010 [63] (Figure 5b) provided insights into optimizing measurement stability. Subsequent layouts, such as the right-angle multi-optical head scheme introduced in 2014 [64] (Figure 5c), and the complex multi-optical head arrangement designed specifically for wafer stages in lithography in 2019 [65] (Figure 5d), further refined these principles, highlighting the progressive strides made in constructing stable, high-resolution multi-DOF measurement frameworks.

In addition, Lee et al. [66] introduced an optical encoder-based system capable of measuring six-DOF motion errors in ultra-precision stages with enhanced accuracy, achieving angular error resolutions below 0.03 arcsec and displacement resolutions down to 0.4 nm. Its validated results closely match those from autocollimator measurements. However, its design still employs differing optical paths for various measurement targets, leading to inconsistent environmental responses that hinder high-precision real-time dynamic measurement. To tackle these limitations, researchers have also explored strategies to mitigate crosstalk errors in multi-DOF measurement. For example, Matsukuma et al. [67] investigated methods to reduce such errors, thereby improving measurement stability and accuracy. Collectively, these studies and evolving design philosophies illustrate the continuous refinement of multi-optical-head approaches, progressively overcoming inherent challenges and paving the way toward more robust, versatile, and precise multi-DOF measurement systems.

## 3. Laser Interferometry

**Figure 6:**
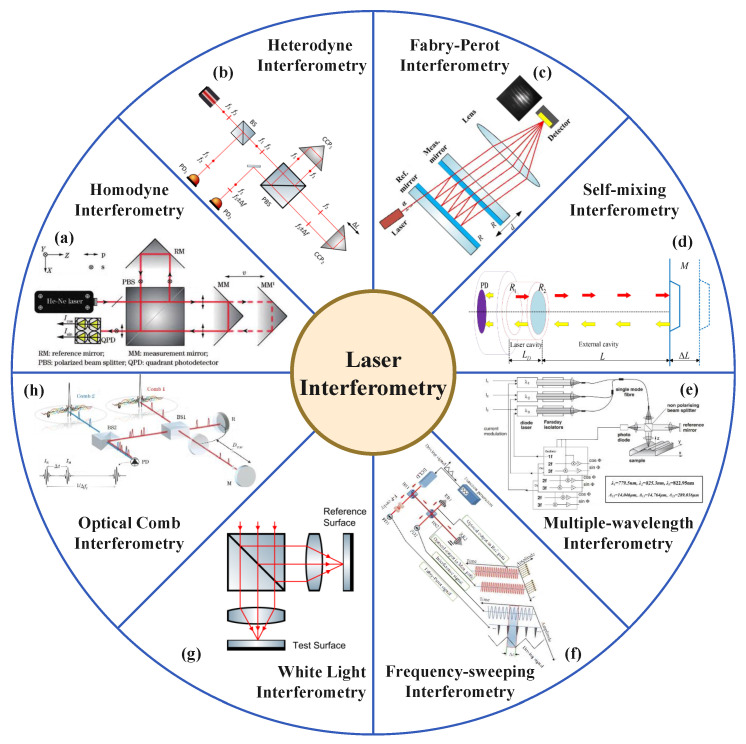
Key techniques in laser interferometry: (**a**) Principle diagram of homodyne laser interference measurement, reprinted from [68]; (**b**) Schematic diagram of two-frequency heterodyne interferometry; (**c**) Principle diagram of Fabry–Perot interferometry, reprinted from [69]; (**d**) Structure of the three-cavity F–P model for self-mixing interferometry, reprinted with permission from [70], copyright 2021 Elsevier; (**e**) Schematic diagram of a profilometry with a multi-wavelength diode laser interferometry, reprinted with permission from [71], copyright 2004 IOP Publishing; (**f**) Schematic of laser ranging of frequency-sweeping interferometry, reprinted from [72]; (**g**) Schematic diagram of a white light interferometry; (**h**) Schematic diagram of a dual-comb interferometry, reprinted from [73].

Laser interferometries have been widely employed in precision measurement applications due to their high sensitivity and accuracy in determining displacement, length, and other physical quantities. The principle of laser interferometry is based on the interference of coherent light waves. By splitting a laser beam into two paths—one acting as a reference and the other as a measurement beam—changes in the path length difference between these two beams result in a phase shift, which can be precisely detected as interference fringes. These phase shifts directly correspond to changes in the optical path, allowing for accurate measurements at the nanometer scale or even below. The key techniques of laser interferometry are concluded in Figure 6. The comparison in terms of advantages and challenges of these techniques are concluded in Table 1.

### 3.1. Homodyne Interferometry

**Figure 7:**
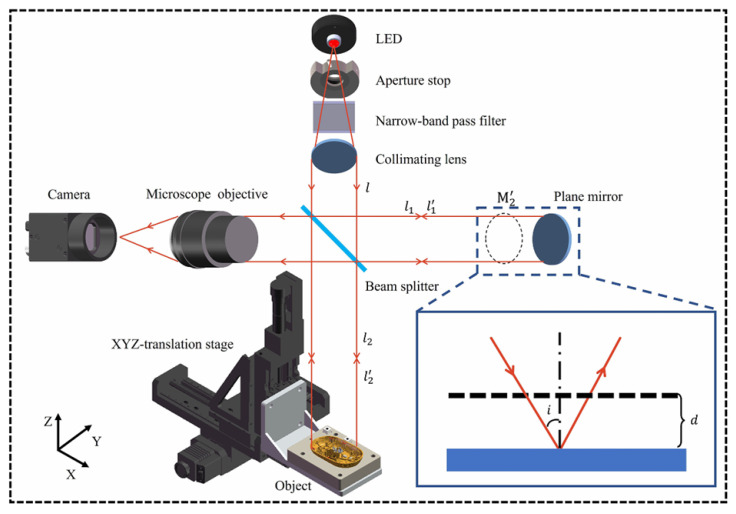
Schematic diagram of a homodyne interferometry, reprinted with permission from [75], copyright 2024 IOP Publishing.

The homodyne interferometry is one of the simplest interferometry configurations [74], relying on variations in light intensity to measure displacement. The Michelson interferometry represents the fundamental structure of a homodyne interferometry. The basic structure is shown in Figure 7. The light is divided into a measurement beam and a reference beam by a beam splitter. The reflected light interferes at the receiver, and the target displacement information can be measured by the change in interference intensity.

Laser homodyne interferometries are widely used in precision measurement due to their simple setup, compact structure, and low sensitivity to the movement speed of the target, offering advantages such as low cost, high precision, and high sensitivity, which have attracted significant research interest. The interference signal in laser homodyne interferometry is a DC amplitude-modulated signal, typically amplified by a DC amplifier. However, the use of a single-frequency laser makes the photodetector vulnerable to interference from ambient light, leading to signal drift. Environmental vibrations also affect the stability of the superimposed light intensity, negatively impacting the electrical signal after photoelectric conversion and causing measurement errors [76]. When the signal amplitude decreases by half, the signal-to-noise ratio (SNR) is significantly reduced due to the drop in fringe contrast, potentially leading to the failure of single-frequency laser interferometries. These issues limit the application of single-frequency laser interferometries in high-precision measurements, especially in harsh environments [77].

As a precise measurement tool based on light intensity detection, the homodyne interferometry is primarily affected by three types of errors in achieving high-speed, high-resolution measurements: periodic error, DC offset error, and non-orthogonal error [78]. Periodic errors, typically caused by the non-ideal characteristics of optical components, lead to periodic fluctuations in the measurement signal within the interferometric fringe period. DC offset errors result from incomplete elimination of the DC component in the signal, causing a constant bias in the measurement, often due to ambient light interference, detector zero-point drift, or electronic noise. Non-orthogonal errors occur when the two signals that should be orthogonal in the interferometry are no longer perfectly orthogonal due to polarization crosstalk or optical path deviations, commonly seen in systems using polarized light interference. These three errors significantly impact the measurement accuracy of homodyne interferometries, making the study of methods for their elimination and compensation crucial.

To reduce DC offset errors, various improvement methods have been proposed. For example, replacing the polarization beam splitter (PBS) with a Wollaston prism with a higher extinction ratio can significantly reduce DC offset errors while effectively compensating for periodic errors [79,80]. Considering the high sensitivity of homodyne interferometries to environmental disturbances, another method to reduce DC offset error involves adding a reference interferometry sharing the same beam path as a compensator, improving the system’s anti-interference capability [76].

To suppress periodic errors, the measurement resolution of homodyne interferometries has reached sub-nanometer levels in recent years with the application of optical path multiplication, electronic frequency doubling, and interferometric fringe subdivision techniques [81,82]. Pisani et al. [83] further enhanced the resolution by employing multiple reflections between two quasi-parallel mirrors, achieving sub-picometer measurement accuracy.

Regarding non-orthogonal errors, Keem.T established a nonlinear error model incorporating the entire optical path in 2004, specifically addressing homodyne systems with four-channel detection units [84]. However, except for the PBS, other optical components were simplified as ideal models. They identified the non-ideality of the PBS as the main source of non-orthogonal and DC offset errors. Subsequently, in 2008, Pisani.M proposed a homodyne laser interferometry with a folded optical path structure, reducing the wavelength of the laser by a factor of 1/N through N-fold path folding, thereby simultaneously reducing both nonlinear and periodic errors to 1/N of their original values. Experiments successfully suppressed nonlinear errors to the picometer level through 100-fold path folding [83,85].

**Figure 8:**
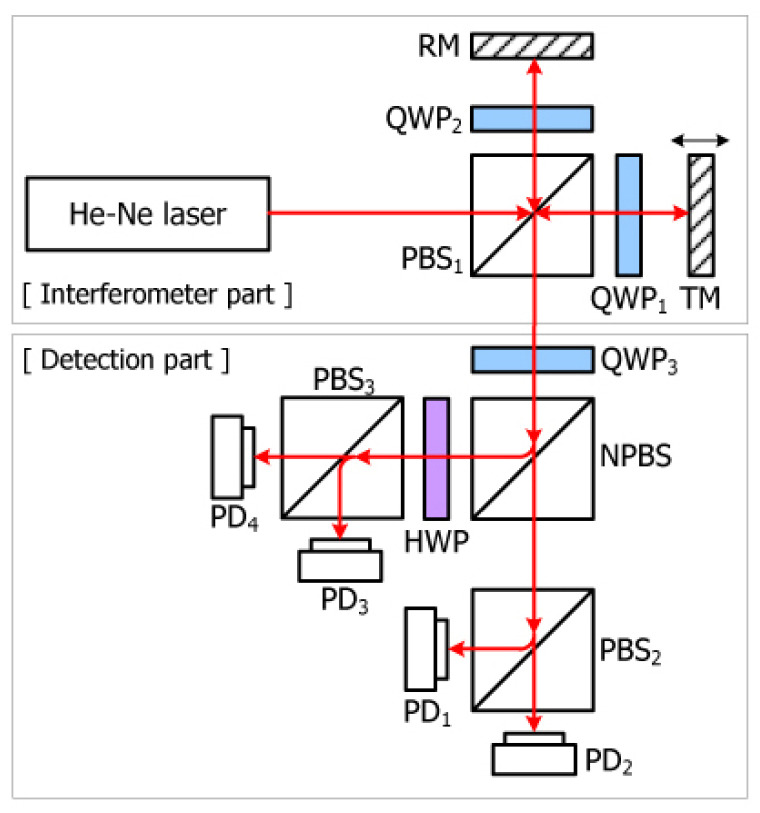
Simple diagram of a homodyne interferometry with quadrature detection system, reprinted from [86].

In 2009, Kim.J introduced parameters related to the slow axis direction of wave plates in the nonlinear error model, analyzing the impact of PBS performance non-idealities and polarization crosstalk caused by wave plate angles on non-orthogonal errors, as shown in Figure 8. Using a gain adjustment method, they effectively suppressed non-orthogonal errors by adjusting the angles of multiple wave plates in the optical path [86]. To further reduce non-orthogonal errors, Dobosz.M from Warsaw University of Technology in Poland proposed a homodyne laser interferometry structure based on fringe detection in 2012. By converting planar interference fringes into equal inclination fringes using a birefringent wedge and detecting positions with a 90° phase difference using an array photodetector, they successfully avoided non-orthogonal errors caused by polarization crosstalk in traditional optical paths [87].

In 2015, Professor Hu Pengcheng’s team from Harbin Institute of Technology developed a nonlinear error model for a four-channel detection unit optical path to address the issue of DC bias variation due to changes in measurement beam intensity and proposed a homodyne laser interferometry structure for DC bias suppression [79]. In 2016, Professor Cui Junning’s team focused on eliminating non-orthogonal errors by redesigning the interferometric unit structure to avoid polarization crosstalk, effectively suppressing non-orthogonal errors [82]. Finally, in 2017, Professor Chen Benyong’s team from Zhejiang Sci-Tech University proposed a dual homodyne laser interferometry based on phase modulation, aiming to minimize nonlinear and non-orthogonal errors caused by polarization crosstalk by avoiding the use of polarization-sensitive optical elements, such as PBS and wave plates, thus achieving higher precision in laser interferometry [88].

### 3.2. Heterodyne Interferometry

The heterodyne dual-frequency interferometry technique is based on the principle of combining two high-frequency sinusoidal waves with a small frequency difference to generate a heterodyne beat frequency signal for displacement measurement. The frequency of the beat signal is exactly the difference between the two sinusoidal wave frequencies, referred to as the heterodyne frequency. If the phase of one of the sinusoidal waves changes, the phase of the beat signal also changes correspondingly, thereby preserving the phase information of the original signal. Heterodyne interferometry is significant for practical engineering because it extracts the phase variation of high-frequency signals, which are difficult to measure directly, through the low-frequency beat signal, greatly simplifying the subsequent precise phase detection process.

**Figure 9:**
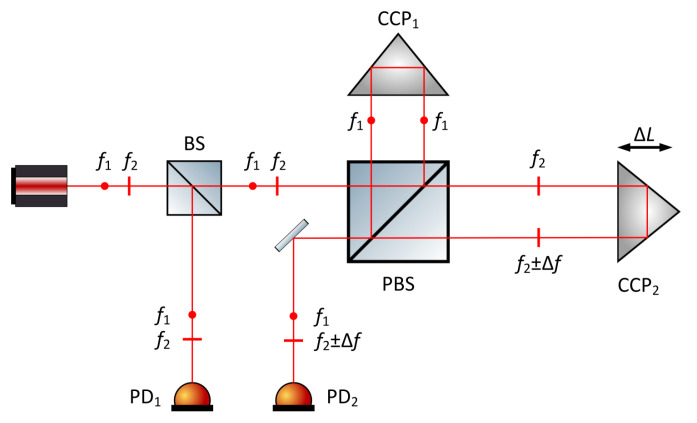
Schematic of a heterodyne interferometry with two orthogonally polarized modes.

The basic measurement principle of heterodyne interferometry is illustrated in Figure 9: the laser output consists of two orthogonally polarized components, S light and P light, with frequencies of f1 and f2, respectively. The frequency difference between the two is achieved through the Zeeman effect [89] or acousto-optic modulators (AOMs) [90,91]. After passing through the beam splitter (BS), the output light is divided into two beams, serving as the reference and measurement beams. The reference beam interferes at the reference photodetector to generate the reference beat signal. The measurement beam, upon passing through the polarization beam splitter (PBS), reflects the S-polarized light and transmits the P-polarized light. The two beams of polarized light are modulated by AOMs and then recombine at the PBS and interfere at the measurement photodetector, generating the measurement beat signal. By demodulating the phase difference between the two beat signals, the displacement information of the measurement mirror can be obtained.

Compared to single-frequency laser interferometries, dual-frequency laser interferometries exhibit significant advantages in terms of the signal-to-noise ratio (SNR), anti-interference capability, and signal processing. The signal of a single-frequency laser interferometry is a DC amplitude-modulated signal, which is susceptible to drift in detectors and signal processing circuits, resulting in low SNR and weak anti-interference ability. In contrast, the signal of a dual-frequency laser interferometry contains differential terms, and other DC components are effectively suppressed during signal processing, leading to higher SNR, stronger anti-interference capability, higher resolution, and simpler signal processing. Additionally, dual-frequency laser interferometries offer a wider dynamic measurement range and can be directly traced to length standards, making them widely applicable in fields such as ultra-precision equipment manufacturing and precision metrology.

**Figure 10:**
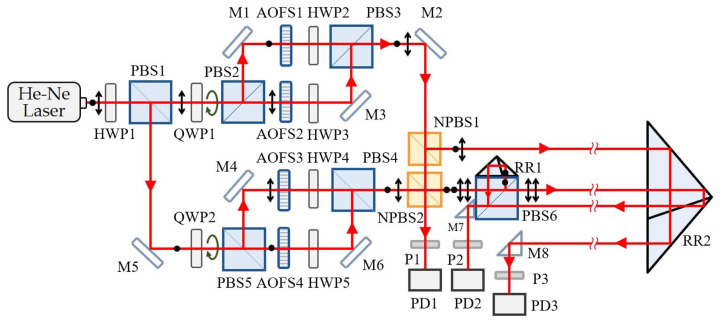
Principle diagram of a superheterodyne interferometry, reprinted from [92].

Laser superheterodyne interferometry represents a further advancement of laser heterodyne interferometry. Its core principle involves performing a secondary heterodyne process on two heterodyne signals, as shown in Figure 10. During this process, the twice-heterodyned signal is converted into an electrical signal, processed through a signal conditioning circuit, and input into a phase meter to acquire phase difference information, ultimately yielding the measurement result through computation. The primary advantage of this technique is that it avoids the need for separate detection of longitudinal mode signals in heterodyne interferometry, effectively eliminating errors caused by mode coupling. Moreover, by incorporating an acousto-optic frequency shifter (AOFS) into the heterodyne interferometry system, the frequency difference of the laser can be effectively reduced, which not only lowers the detection requirements of the photodetector but also simplifies the complexity of subsequent signal processing, thereby significantly improving the speed and accuracy of measurements.

Heterodyne interferometry technology has seen significant advancements in recent years with the progress of electronic phase measurement technology, and its displacement measurement resolution has reached sub-nanometer levels [93]. Commercial interferometries using helium-neon lasers as the light source offer an optical resolution of λ/2, but with fringe interpolation techniques, displacement measurement resolution can be further improved to λ/2048 (approximately 0.31 nm) [94]. However, the presence of periodic nonlinear errors, such as the influence of mixed-frequency sources and leakage in polarization components, can reduce the coherence of the interferometric signal and introduce additional phase variations, which pose significant challenges to improving measurement accuracy. Most commercial laser interferometries exhibit periodic nonlinear errors in the range of several nanometers to tens of nanometers, which are critical for precision enhancement. To address this, researchers have developed algorithms [95,96] or employed spatially separated polarized beams [97] to reduce such errors. Joo et al. [98] used two spatially separated beams with a frequency difference to prevent polarization mixing and generated beams of different frequencies from a stable single-frequency light source using an acousto-optic frequency shifter.

In 2011, Youngkyu et al. designed a light heterodyne measurement system based on acousto-optic modulators (AOMs) that includes two signal paths and successfully measured small vibrations [90]. That same year, Jonathan D. et al. used two AOMs to modulate the light beams, decompose these beams into two linearly diffracted beams, and recombine them into a reference beam, which was coupled into polarization-maintaining fibers to form the measurement beam. They developed a fiber-coupled micro-displacement measurement system with no periodic or linear correlation errors [99].

**Figure 11:**
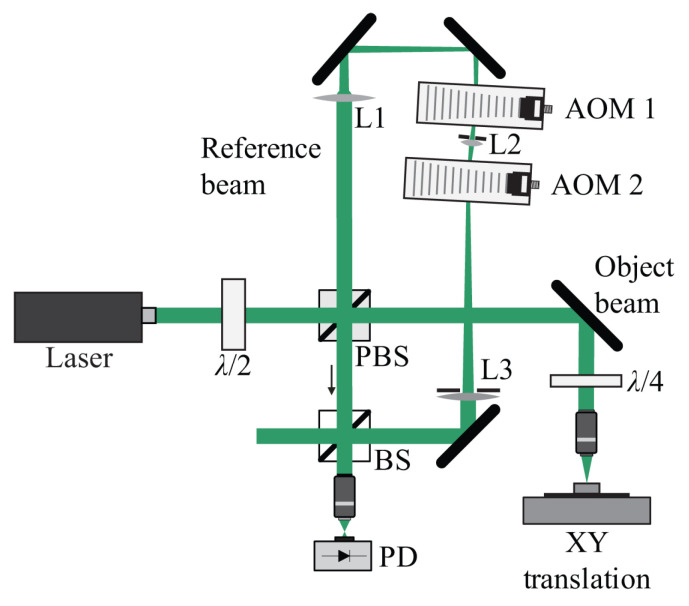
Optical configuration of the heterodyne laser interferometry with femtometer sensitivity, reprinted from [100].

In 2013, Erlend Leirset et al. developed a light heterodyne measurement system capable of detecting micro-vibrations in the range of 100 kHz to 1.3 GHz, with enhanced amplitude sensitivity. This system utilized an AOM for frequency shifting, extending the frequency range while effectively reducing the impact of beam deflection on the laser frequency [100]. The optical setup is illustrated in Figure 11.

In 2015, Hao Yan et al. proposed a dual-heterodyne laser interferometry for simultaneous measurement of linear and angular displacement. The system employed a phase measurement method based on cross-correlation analysis and utilized a PXI-bus data acquisition system to suppress common-mode noise. Experimental results showed that the noise levels for linear and angular displacement were approximately 1 pm/Hz^1/2^ and 0.5 nrad/Hz^1/2^, respectively, under 1 Hz conditions [101]. That same year, Minhao Zhu et al. developed a DRT F-P system for absolute displacement measurement, achieving a displacement measurement resolution of 10 pm by locking two external-cavity diode lasers onto the resonances of a high-finesse Fabry–Pérot cavity [102].

In 2016, Hung-Lin Hsieh et al. constructed a high-resolution angular displacement measurement system that combined common-path technology and the birefringent effect in a heterodyne interferometry design, ensuring high accuracy over a wider angular range [103]. That same year, Bin Sun et al. proposed a new error compensation model, significantly reducing the measurement error due to incident angle variation in a laser displacement sensor, and demonstrated efficiency superior to that of a high-precision coordinate measuring machine (CMM) in measuring gas turbine blades [104].

In 2020, Ki-Nam Joo et al. designed a compact heterodyne interferometry that achieved picometer-level displacement measurement sensitivity with good thermal stability [105]. That same year, Dong N. T. et al. proposed a heterodyne interferometry system incorporating a phase-locked loop algorithm, achieving 10 pm-level mechanical displacement measurements in both air and vacuum environments [106].

In 2022, Dong N. T. et al. developed a heterodyne interferometry system for sub-micrometer mechanical displacement measurement. The system comprised a dual-path heterodyne interferometry using a Zeeman-stabilized helium-neon laser as the light source and a custom single-phase-locked loop phase meter. Since the beat frequency of the dual-path heterodyne interferometry was in the MHz range, down-mixing technology was employed to process the heterodyne signals, allowing for the use of a low-cost, low-sampling-rate ADC. Displacement measurements were performed using both the reference phase meter and the custom phase meter, yielding a mechanical displacement of approximately 2.6 nm. Combined with down-mixing technology, sub-nanometer mechanical displacement measurements were achieved [107].

### 3.3. Sinusoidal Phase Modulation Interferometry

Sinusoidal phase modulation (SPM) interferometry is a modification of the Michelson interference structure. By applying a high-frequency carrier to the reference light for phase modulation, the low-frequency signal to be measured is transferred to the sideband of the high-frequency carrier signal, and then the phase information of the interference signal is obtained by phase carrier demodulation technology, thereby achieving length measurement. Compared with homodyne interferometry, it has improved anti-interference ability; compared with heterodyne interferometry, the optical path is simpler and the frequency aliasing is avoided. In the SPM interferometry system, the reference light is phase-modulated by internal modulation or external modulation. Internal modulation is to change the laser frequency directly by changing the driving voltage of the laser. External modulation is to add a modulation device, such as piezoelectric ceramics (PZT) and electro-optical phase modulators (EOM), to the reference arm of the optical path. In 1988, Sasaki O. et al [108] proposed a SPM interferometry based on PZT modulation to measure the surface profile of a diamond-turned aluminum disk with a measurement accuracy of several nanometers. In 1990, they proposed the internal modulation method [109], which modulates the output wavelength by adjusting the current of the laser diode, and eliminates external interference through feedback control. The measurement repeatability can reach less than 1 nm. In PZT modulation, mechanical vibration will affect the stability of the measurement system, and the speed of the measured target is also limited by the PZT modulation frequency. In the internal modulation based on current modulation, the laser power will also fluctuate due to the modulation, thereby introducing measurement errors. To this end, Minomi U. [110] proposed an SPM interferometry based on EOM high-frequency modulation in 1991. The EOM is placed in the reference arm to modulate the reference light. When the speed of the target to be measured is 1 m/s, a measurement accuracy of 80 nm can be achieved. Since EOM can greatly increase the frequency of the modulation signal, it is more widely used in the SPM interferometry.

**Figure 12:**
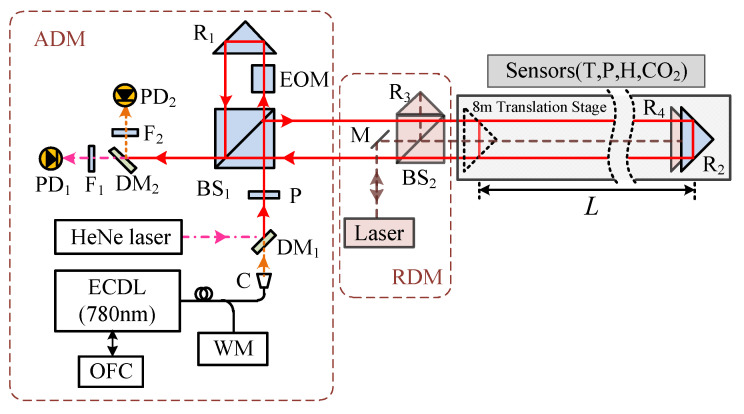
Schematic diagram of the SPM absolute length measurement interferometry, reprinted from [111].

In order to apply the SPM interferometry to achieve absolute length measurement, Shihua Zhang et al. [111] proposed an SPM absolute length measurement interferometry combining frequency-sweeping interferometry (FSI) and multi-wavelength interferometry (MWI) in 2018. Its structure is shown in Figure 12. He performed frequency sweeping for coarse measurement by FSI, and frequency hopping for fine measurement by MWI. The laser wavelength is calibrated by an optical frequency comb. A step of 3 µm at a distance of 8.25 m was measured in a range of 15 µm, which achieved a standard deviation of 0.29 µm of the dual of −0.40 µm. In 2022, he proposed an FSI with reference interferometry based on SPM interferometry for absolute length measurement [112]. By compensating the influences of drifts and applying linear regression to obtain the ratio of interference phase changes between the measurement and reference interferometry, the measurement uncertainty can be reduced to 10^−5^.

There are three main methods to obtain the measured phase in the interference signal, namely, the Fourier analysis method [108], the phase locked loop demodulation method [111], and the phase generation carrier (PGC) demodulation method. Among them, the PGC demodulation method is widely used due to its advantages, such as high sensitivity, large dynamic range, good linearity, and strong anti-interference ability [113,114,115]. In the PGC demodulation method, the interference signal needs to be mixed with the carrier signal and its harmonic signal and then low-pass-filtered to obtain a pair of orthogonal components related to the measured phase. The PGC demodulation method can be divided into the PGC-DCM (Differential-Cross-Multiplying) method [116] and the PGC-Acrtan (Arctangent) method [117]. The PGC-DCM method is greatly affected by the intensity of the light beam and the visibility of the interference fringes; the PGC-DCM method is not affected by the light intensity disturbance, but requires phase unpacking operation, and resists harmonic distortion. In addition, both methods are greatly affected by the phase modulation depth and carrier phase delay. In order to solve the nonlinear errors introduced by the phase modulation depth and carrier phase delay, many researches have been conducted to improved the conventional PGC method.

**Figure 13:**
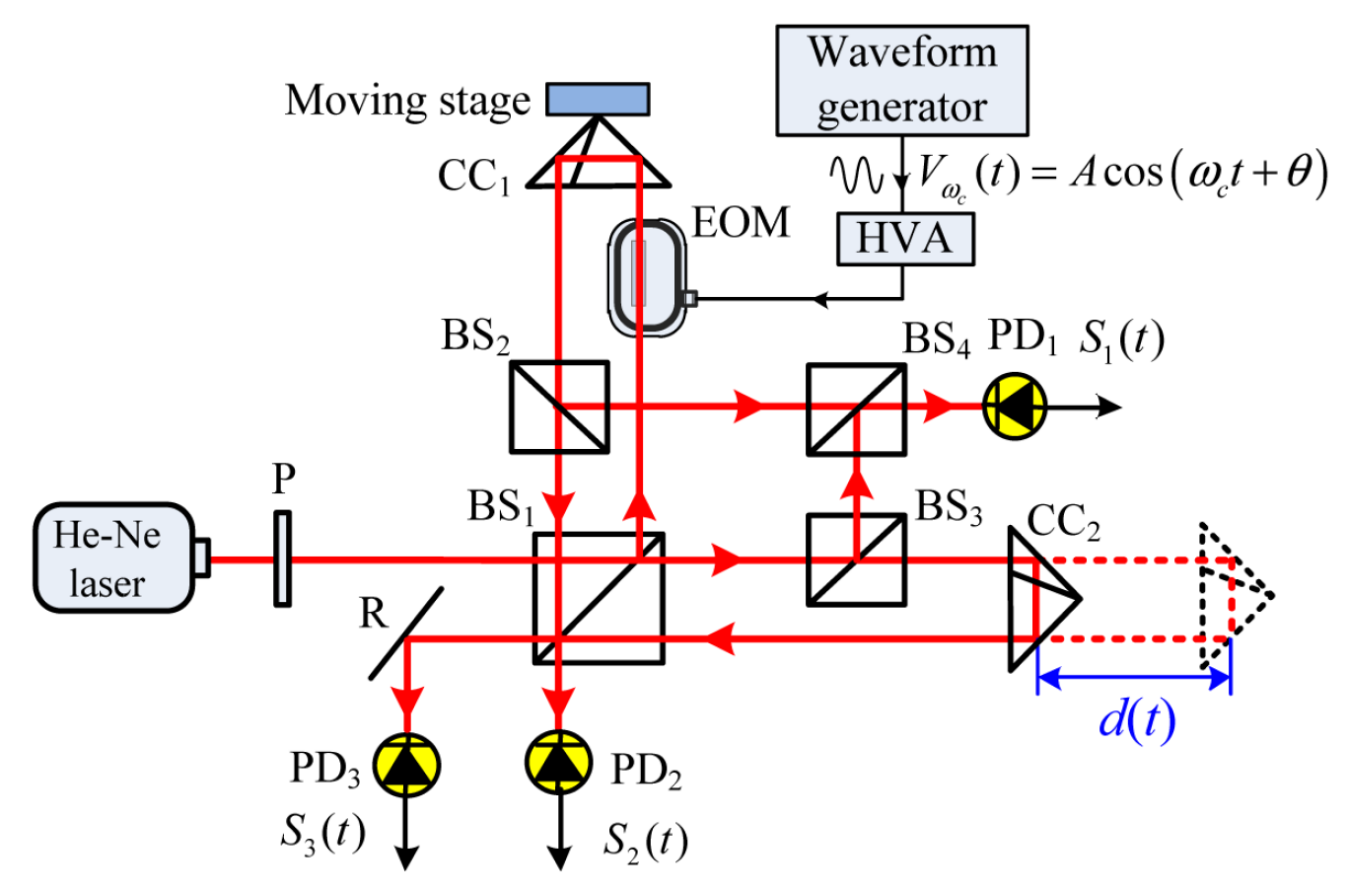
Schematic of the modified EOM-based SPM interferometry, reprinted from [122].

Jun He et al. proposed an ameliorated PGC demodulation algorithm based on an arctangent function and differential-self-multiplying. This algorithm reduces the influence of light intensity fluctuation, and the demodulation result is no longer affected by the modulation depth. The total harmonic distortion is reduced to 0.1%, and the signal-to-noise and distortion is increased to 60 dB, but nonlinear errors are introduced in the solution process [118]. In 2015, Shuai Zhang et al. proposed the PGC-RCM algorithm, which can eliminate the influence of light intensity disturbance and effectively demodulate the acoustic signal by introducing a reference signal [119]. This method improves the signal-to-noise ratio by 11.78 dB compared with the PGC-DCM algorithm, but it has high requirements on the filter performance. Ailing Zhang et al. proposed an interferometric sensor with asymmetric division and a differential-self-multiplication phase-generated carrier (PGC-AD-DSM) demodulation algorithm based on fundamental frequency mixing. The signal-to-noise and distortion ratio of the sensor with the proposed algorithm achieves a gain of 7.77 dB over the PGC-Arctan algorithm and 9.48 dB over the PGC-DCM algorithm [120]. In 2017, Shihua Zhang et al. proposed a real-time phase delay compensation method by regulating a compensating phase introduced to the carrier to maximize the output of the low-pass filter so as to make the carrier synchronize with the interference signal [121]. Nanometer accuracy was realized by using the proposed phase delay compensation method. Later in 2018, he realized the real-time normalization in PGC demodulation and proposed a fixed-phase-difference detection method to evaluate periodic nonlinearity [122]. The modified structure is illustrated in Figure 13.

### 3.4. Fabry–Perot Interferometry

The Fabry–Perot (FP) interferometer typically consists of two highly reflective mirrors, forming an optical resonant cavity where multiple beam interference occurs as the laser light reflects back and forth between the mirrors. When the distance between the two mirrors is fixed, the device is referred to as an FP etalon. If the distance can be adjusted, it is called an FP interferometer. Due to its multiple-beam interference effect, the interference fringes produced by the FP interferometer are finer and brighter compared to those generated by two-beam interferometers. Furthermore, because the reference and measurement beams share the same optical path, the system is not affected by the reference arm [69].

**Figure 14:**
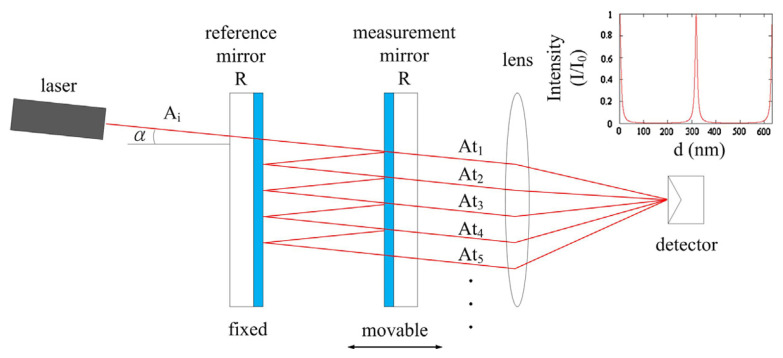
Schematic of a Fabry–Perot interferometry, reprinted with permission from [125], copyright 2013 Elsevier.

As illustrated in Figure 14, the classical FP interferometry is based on the principle of multiple-beam interference and consists of an optical resonant cavity formed by two parallel, adjustable highly reflective mirrors. When a coherent beam of light enters the FP cavity, part of the light reflects back and forth inside the cavity, creating multiple-beam interference. Inside the FP cavity, the transmitted light beams, resulting from the repeated reflections of the incident beam, interfere with each other. The wavelength that fulfils the FP condition will be output from the FP cavity. The FP condition can be described by a simple formula: a multiple of half of the vacuum wavelength equals the cavity length. It should be noted that the FP interferometer involves multipath interference and is highly complex, and the model shown in Figure 14 is simplified. The highly reflective mirrors in the FP cavity are commonly based on Distributed Bragg Reflectors (DBRs). DBR is a mirror structure composed of alternating stacks of thin films with low and high optical refractive indices. In real conditions, the modes would penetrate into the dielectric mirrors, causing a deviation in the distance between the two central interfaces of mirrors. Furthermore, when considering the movement of the DBR, the influence of the electro-drive on the internal membrane of the DBR will also affect the tuning efficiency [123,124].

In the field of high-precision micro-displacement measurement, the frequency tracking method is usually applied. This method converts displacement measurements into frequency variations, enabling precise displacement detection. In practice, the technique first locks the tunable laser’s frequency to the peak output of the optical resonant cavity, while the photodetector continuously monitors this peak. When the measurement mirror moves, the photodetector detects changes in the resonance peak, which is then controlled by a frequency-modulation servo system (FMSS) to ensure that the laser frequency remains aligned with the maximum output of the resonant cavity. By recording the cavity length *L* and the frequency shift Δf of the laser, and using the initial frequency *f*, the displacement of the mirror can be calculated. The frequency shift Δf is measured from the beat signal between the tunable laser and a frequency-stabilized laser. This technique is widely used in precise micro-displacement measurement due to its high measurement accuracy and resolution. However, the system is highly complex, requires precise craftsmanship, and has a relatively small measurement range and high cost.

The FP interferometry, with its shared reference and measurement beam paths, exhibits low sensitivity to environmental disturbances. However, one major challenge faced by traditional FP interferometries is the limitation on measurement range when the reference and measurement mirrors are not perfectly parallel. To address this issue, Rabinowitz [126] replaced the traditional measurement mirror with a corner cube reflector (CCR) and used a folded cavity structure, which not only corrected misalignment issues but also improved measurement resolution to λ/16. In addition, Chang et al. [125] developed an improved folded FP interferometry, which allows the interference pattern to be adjusted by replacing glass plates with different reflectivities and by adjusting the tilt angle of the coated plane mirrors to match the fringe spacing with the detector’s sensing area. Experimental results show that this design achieves a measurement range of over 100 mm with sub-wavelength measurement accuracy.

Further optimization of the FP interferometry includes advancements in the light source, such as the use of widely tunable fiber lasers to expand the measurement range [127], and the application of fiber-optic technology to achieve a more compact FP interferometry design [128].

### 3.5. Self-Mixing Interferometry

Laser self-mixing interferometry (SMI) is a precise measurement technique based on the laser self-mixing interference effect. This effect occurs when a portion of the laser beam, after illuminating the surface of a target object, is scattered or reflected back into the laser resonant cavity, coupling with the intracavity optical field and modulating the laser’s output power and frequency. The core principle is the interaction between the intracavity optical field and the feedback optical field from the external cavity object, resulting in a unique multiple-beam interference effect.

**Figure 15:**
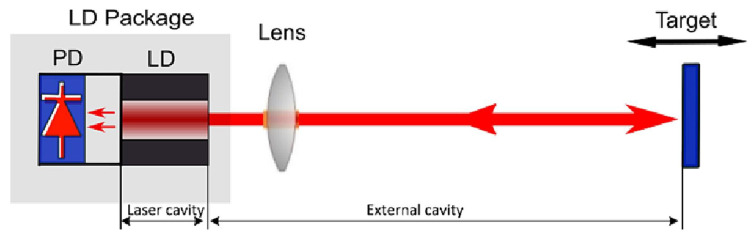
Self-mixing interferometry experimental setup, reprinted from [129], copyright 2024 Elsevier.

In SMI, the stability of the laser must be maintained within both the intracavity and the external cavity involving reflections or scattering. As illustrated in Figure 15, during the measurement process, the light emitted by the laser passes through the lens into the external cavity, where it illuminates the target object. A portion of the reflected light re-enters the laser cavity and interferes with the intracavity laser light. This interference effect is captured by the photodetector, which detects the modulation in the laser’s amplitude. The modulation originates from the object’s response to the laser movement, causing oscillations in the output laser power, with the oscillation period corresponding to half of the wavelength of the object’s displacement (λ/2). By analyzing the detected interference signal, the system can measure the displacement of the object with high precision.

Compared to traditional two-beam interferometries, the interference effect in SMI occurs within the laser cavity itself, eliminating the need for an external reference path, which makes the system more compact. This advantage makes SMI particularly suitable for measurements in complex and confined spaces [130]. Since any reflection from the object that re-enters the diode cavity modulates the laser output, SMI typically employs low-cost, compact diode lasers [131] as the light source. Shinohara et al. [132] proposed a method for distance measurement using a self-mixing laser diode. The interferometry detects the backscattered light from the object and measures the average mode-hop interval of continuous external mode hops. This system achieved high-precision measurements with an error of ±0.15% in the 0.2∼1 m range, demonstrating the potential of diode lasers for their high sensitivity and power, while the simple interferometry configuration effectively reduces laser power losses. Since this work, the laser feedback effect in diode lasers has been widely applied to absolute distance measurement.

The laser feedback effect in diode lasers refers to the near-linear response of the laser’s operating frequency to small changes in the driving current [133]. Utilizing this characteristic, high-precision length measurements can be achieved by periodically modulating the driving current of the laser to scan the output frequency. Kou et al. [134] developed an SMI system based on a vertical-cavity surface-emitting laser (VCSEL), which achieved precise control of the laser output frequency through shaped modulation of the injection current. The system used a sawtooth-shaped tuning current to drive the VCSEL, effectively reducing the nonlinearity of the current during tuning and improving the resolution of distance measurements. It achieved a resolution of 20 microns within a range of 2.4∼20.4 cm. Moench et al. [135], Michalzik [136], and Gouaux et al. [137] also developed VCSEL-SMI using driving current modulation.

### 3.6. Multi-Wavelength Interferometry

In single-wavelength interferometry for length measurement, the phase ambiguity is 2π, resulting in an unambiguous distance of λ/2. This means that when the measured distance exceeds λ/2, fringe counting is required for accurate length measurement. In such cases, to ensure measurement continuity, the laser beam needs to continuously track the target object. If tracking is interrupted, the measurement process must restart. To address the range limitation of single-wavelength interferometry, multi-wavelength interferometry combines interference fringes from different wavelengths to form a longer synthetic wavelength, reducing phase ambiguity and enabling precise measurement over longer distances.

In an interferometry system with a wavelength of $\lambda_{1}$, whether using zero-path interferometry or heterodyne interferometry, the distance *L* can be uniformly expressed as

(2) $$L=\frac{\lambda_{1}}{2}\left(m_{1}+e_{1}\right)$$

where $m_{1}$ is the unknown integer multiple of the 2π phase, and $e_{1}$ is the fractional phase obtained from the phase measurement value $\varphi_{1}$ (in radians), satisfying the conversion relationship $e_{1}=\varphi_{1}/2\pi$. If another laser wavelength is introduced for interferometry, the distance *L* can be expressed as

(3) $$L=\frac{\lambda_{2}}{2}\left(m_{2}+e_{2}\right)$$

By solving these equations simultaneously, the distance *L* can be determined as

(4) $$L=\frac{\lambda_{s}}{2}\left(m_{1}-m_{2}+e_{1}-e_{2}\right)$$

where $\lambda_{s}=\lambda_{1}\cdot \lambda_{2}/\left|\lambda_{1}-\lambda_{2}\right|=c/\Delta \nu$, and Δν is the frequency difference corresponding to the wavelengths. The unambiguous measurement range at this point is $\lambda_{s}/2$. As long as the uncertainty $U_{L}$ in the measured distance is smaller than $\lambda_{s}/2$, the values of $m_{1}$ and $m_{2}$ can be uniquely determined.

Multi-wavelength interferometry extends the concept of equivalent synthetic wavelengths by forming a multi-level chain of virtual synthetic wavelengths. Starting from the highest-level synthetic wavelength, the integer fringes of the synthetic wavelengths are determined step-by-step, ultimately using a single wavelength for precise distance calculation. This method not only retains the nanometer-level resolution and high precision of single-wavelength interferometry but also significantly expands the unambiguous distance range by using synthetic wavelengths formed from different interference wavelengths. Multi-wavelength interferometry can achieve high-precision, real-time, and dead-zone-free measurements over ranges of several meters or even tens of meters. The measurement results are traceable, capable of being traced back to microwave frequency standards, making this technique highly valuable for large-scale high-end equipment manufacturing and length metrology [138].

In 1973, Polhemus [138] discussed dual-wavelength interferometry for distance measurement under static conditions. In 1988, Dandliker et al. [139] proposed a heterodyne detection method, enabling high-resolution measurements at arbitrary synthetic wavelengths without requiring interference stability or optical separation at different wavelengths, which was crucial for sub-millimeter resolution distance measurements.

**Figure 16:**
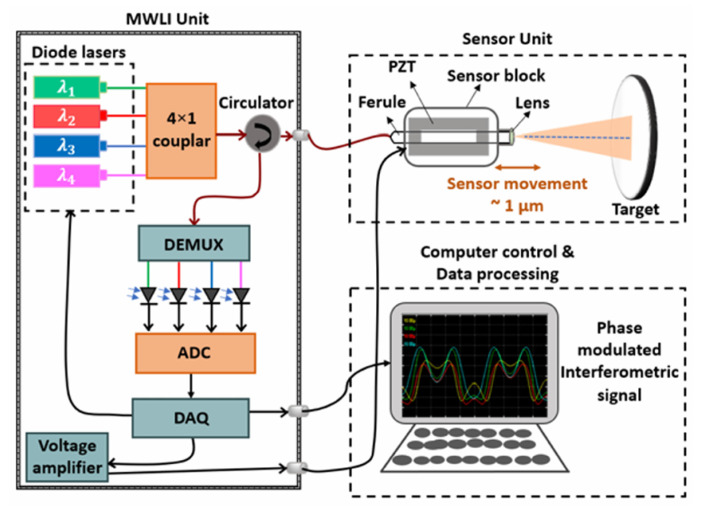
Multi-wavelength interferometry with phase modulation, reprinted from [144].

Although synthetic wavelengths can extend the measurement range, they may also increase measurement uncertainty. To address this, Cheng and Wyant [140] introduced data from a third wavelength to reduce the uncertainty in dual-wavelength interferometry, demonstrating that using more than two wavelengths can achieve low-uncertainty, unambiguous length measurements. Meiners-Hagen et al. [71] developed a three-wavelength interferometry that uses the locking technique of three laser diodes, simultaneously capturing interference signals from multiple wavelengths with a single photodetector. This system performs preliminary measurements over a large synthetic wavelength range $\Lambda_{23}$ (approximately 145 µm) and then determines the fringe order using a smaller synthetic wavelength (14 µm). Additionally, multi-wavelength interferometries can leverage the Doppler effect to measure moving objects [141]. To further enhance performance, researchers have introduced interferometric systems using four or more wavelengths [142,143,144], which further improve measurement accuracy and range, as shown in Figure 16. Meanwhile, simultaneous phase detection in multi-wavelength interferometry is essential for high-accuracy dynamic absolute length measurement. Shihua Zhang [145] proposed a simultaneous phase detection scheme based on a frequency-division multiplexing technique in 2022. Results of simulations showed that the resolution, accuracy, and nonlinear error of the two demodulated phases are all better than 0.01°. Experimental results of 10 nm continuous step displacement measurement in 1 µm indicate that sub-nanometer accuracy and nonlinear error are achieved.

### 3.7. Frequency-Sweeping Interferometry

Frequency-sweeping Interferometry (FSI) is a high-precision length measurement technique that utilizes a linearly tunable laser. A sawtooth wave is used to linearly modulate a tunable laser. The laser beam is split into two paths by a polarization beam splitter: one as the reference beam and the other as the measurement beam. After returning, the two beams produce a beat frequency signal through heterodyne interference, which is processed to obtain distance information. Compared to single-frequency and dual-frequency laser interferometry, FSI effectively resolves the 2π phase ambiguity issue, offering advantages such as no measurement ambiguity, strong anti-interference capability, and the ability to measure non-cooperative targets. Additionally, FSI does not require a guide rail. The absence of a guide rail avoids cumulative errors during counting and improves measurement efficiency, making the measurement of step-like structures possible. Moreover, it eliminates the need to reposition the target after obstructions, addressing the diverse requirements of practical measurements. Unlike multi-wavelength interferometries, FSI relies solely on a tunable laser and a frequency sweep measurement system, without the need for two or more independently stabilized lasers.

The key advantage of FSI lies in generating a synthetic wavelength through frequency modulation, accurately recording the maximum of the synthetic wavelength via the detector, making it particularly suitable for large-range measurements [146,147,148]. However, since FSI is highly sensitive to distance changes during the sweep process, its resolution is typically limited to within a few micrometers. To address this limitation, researchers have proposed various improvements in recent years. For example, Cabral and Rebordao [149] proposed a theoretical model of FSI and an uncertainty evaluation method. To reduce the impact of environmental disturbances, a second laser source is often introduced [150], or algorithms are employed to compensate for movement errors, such as continuous phase measurement [151].

**Figure 17:**
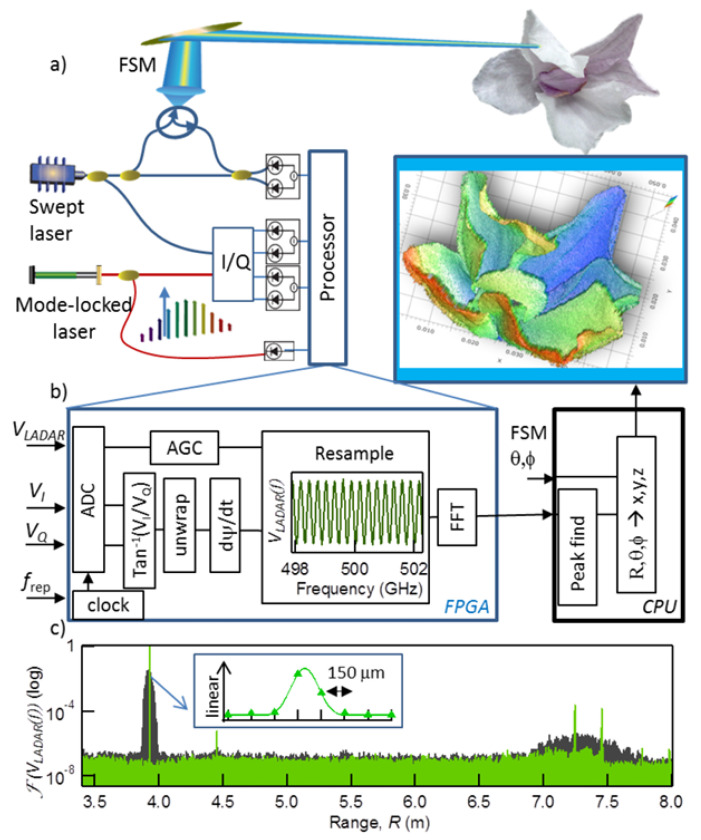
(**a**) Overview of the experiment setup. (**b**) Block diagram of the data processing. (**c**) Linearized range spectrum. Reprinted from [155].

FSI technology has evolved with the development of tunable lasers. In 1986, Kikuta [152] first proposed using injection-current tunable lasers to achieve absolute distance measurement with FSI. The tunable laser used in the experiment had a tuning bandwidth and linewidth of only a few hundred MHz, with a measurement range limited to several tens of centimeters. Since 2009, FSI has made significant advances in frequency modulation control and measurement accuracy. Satyan et al. [153] developed a feedback control system for semiconductor laser sources, improving the measurement resolution to 1.5 mm. Liyama et al. [154] used single-mode vertical-cavity surface-emitting lasers (VCSEL), achieving a spatial measurement resolution of 250 μm. Baumann et al. [155] calibrated the laser’s optical frequency using an optical frequency comb, improving the measurement precision to 10 μm at stand-off distances up to 10.5 m, as shown in Figure 17.

Research on FSI also focuses on expanding bandwidth and improving frequency control precision. In 1996, Liyama et al. [156,157,158] achieved 10 GHz bandwidth control using RF modulation technology, enabling precise control of up to 50 GHz of bandwidth. In 2009, Satyan et al. [153] used current-driven tunable lasers as the light source, achieving broadband frequency chirp control with a photonic feedback loop. They locked the sweep slope and shape to the reference frequency of an external electrical signal, achieving 100 GHz linear scans within 1 ms, with a spatial resolution of 1.5 mm. By 2012, they had achieved 400 GHz linear control [159]. In 2010, Roos et al. [160] used a widely tunable external-cavity diode laser (ECDL) as the light source to build a closed-loop phase-locked system, employing fiber-optic self-heterodyne technology. Experimental results showed that, after linearization, the system’s measurement resolution improved to 31 µm, with a precision of 86 nm over a 1.5 m measurement range. In 2011, Barber et al. [161] used an optical frequency comb with ultra-high precision, accuracy, and stability ($10^{-12}$) as a calibration ruler, tracking the nonlinear error of the ECDL and improving the chirp linearity to 15 ppb. In 2015, Mateo et al. [162,163] proposed using a hydrogen cyanide ($H^{13}C^{14}N$) molecular gas absorption cell to real-time monitor the chirp curve of the ECDL. Using feedback control technology, they achieved frequency control with a linearity of less than 1 ppm. That same year, Qin Jie et al. [164] built a wideband phase-locked system based on an ultra-short delay Mach–Zehnder interferometry for linear frequency modulation control. To eliminate residual phase errors after phase-locking, they used digital phase compensation techniques, increasing the coherence length of the laser by 60 times without sacrificing resolution. Experiments demonstrated that using the phase-locked loop system, a distributed feedback laser (DFB) could achieve 80 GHz frequency control within 8 µs, with a frequency error of less than 55 kHz.

### 3.8. White Light Interferometry

White-light interferometry (WLI) was developed to address the phase ambiguity issue in monochromatic phase-shift interferometry. White-light interferometry, also known as low-coherence interferometry, uses a broadband light source for illumination. Due to its short coherence length, interference fringes only appear in a very limited range, and the zero-order fringe has significantly higher intensity and better contrast than other fringes. Compared to monochromatic light, white-light interferometry does not exhibit phase ambiguity in the vertical direction, allowing for a higher measurement range. Theoretically, the vertical measurement range of white-light interferometry is only limited by the scanner’s travel range and the working distance of the interferometric objective lens [165,166,167,168]. Since only the light source is moving in the scanning process, the system structure for white-light scanning interferometry is basically the same as that of monochromatic phase-shift interferometry and can be used as a displacement or distance sensor [165,168]. This method offers advantages such as a large measurement range, high resolution, and non-contact detection.

**Figure 18:**
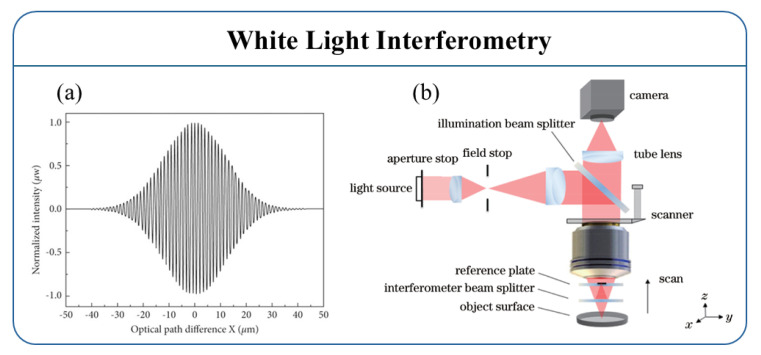
(**a**) The extracted WLI signal, reprinted from [169]; (**b**) Schematic diagram of a Mirau WLI structure, reprinted from [170].

Figure 18a displays the extracted white-light interference signal. Unlike monochromatic interference signals, white-light interference signals typically appear as a cosine envelope modulated by a Gaussian function, and the visibility of the fringes varies with the scanning position. The interference signal reaches its maximum when the optical path difference between the measurement and reference beams is zero. This position corresponds to the coherence peak and represents the relative height information of the corresponding data point on the surface. The combination of all data points’ relative heights forms the overall surface topography of the sample.

A typical white-light interferometry setup includes Michelson, Linnik, and Mirau configurations, with the Mirau-type interferometry structure shown in Figure 18b. The outgoing beam of the broadband light source passes through the Köhler illumination system and the beam splitter, and then enters the pupil plane of the interference microscope objective. After passing through the interferometry beam splitter, it is divided into a reference beam and a measurement beam. The two beams are then, respectively, reflected by the reference plate and the object surface, and then pass through the objective system and the imaging system in turn to form an interference pattern on the camera. The interference pattern is then analyzed by a computer for accurate measurement of the sample’s surface microstructure.

Pavlicek and Hausler [166] developed a fiber-optic distance sensor based on WLI. Since the reference mirror needs to be fully scanned to determine the object’s distance, the measurement speed of white-light interferometry is relatively slow and is only suitable for measuring quasi-static objects. Although WLI’s theoretical measurement accuracy can reach sub-wavelength levels [171], in practical applications, the accuracy is usually limited to within 1 µm due to the signal-to-noise ratio and mechanical drift of the scanner. Additionally, Winarno et al. [172] achieved point-to-point absolute distance measurement using serial low-coherence interferometry, measuring internal distances up to 100 mm with an uncertainty of 178 nm.

Despite the high measurement accuracy and resolution of white-light interferometry, various errors arise from the interference image during the measurement process, such as resolution errors caused by the Rayleigh diffraction limit and light intensity loss due to diffuse reflection. These errors result in typical measurement deviations, such as the batwing effect. The batwing effect refers to the shape error that appears like “bat wings” when measuring steps with a white-light interferometry. Researchers have proposed various compensation and elimination methods to address these errors, including hardware improvements and algorithmic optimization. For example, Niehues [173,174] suggested using dual-wavelength light sources and adding a confocal aperture at the light source to reduce errors. Xie [175] optimized the system by changing the light source wavelength, while Abraham [176] chose appropriate wavelengths and objective lenses and used bandpass filters to suppress the multipath white-light interference signal. Additionally, Benedikt [177] proposed modifying the reference surface’s topography to infer the shape information of the object under test. In terms of algorithms, combining phase-shifting and envelope methods effectively eliminates the batwing effect for relatively smooth surfaces, though this method is not suitable for periodic structures like gratings [178].

### 3.9. LIGO Interferometer

The Laser Interferometer Gravitational-Wave Observatory (LIGO) is a key example of using optical interferometry to detect very small changes in spacetime. It aims to measure gravitational waves, which are tiny distortions in spacetime predicted by Einstein’s theory of general relativity. LIGO’s main setup is similar to a long Michelson interferometer with two arms, each several kilometers in length. A stable laser beam is split into two paths that travel down these arms, reflect off suspended mirrors, and return to be combined. When a gravitational wave passes, it slightly changes the effective length of the arms. This alters the interference pattern of the combined beams, allowing LIGO to measure length changes as small as about $10^{-19}$ m. Such precision can detect signals from events like black hole or neutron star mergers.

**Figure 19:**
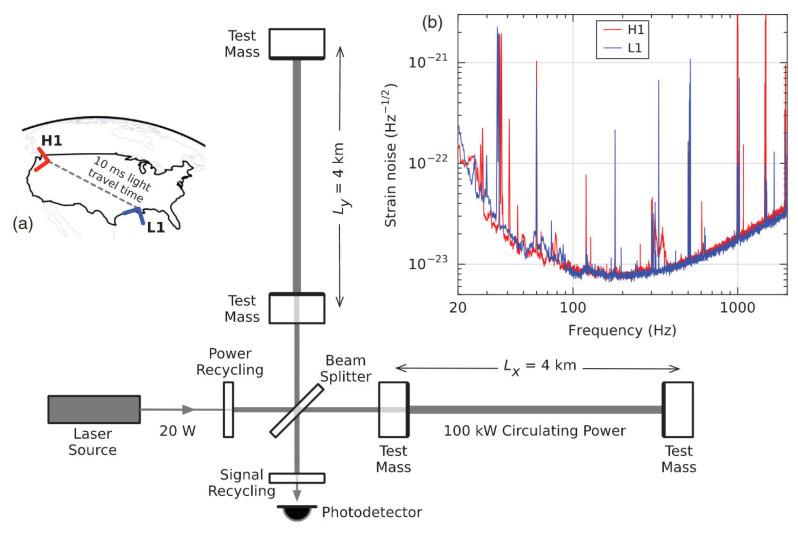
Simplified diagram of an Advanced LIGO detector. (**a**): Location and orientation of the LIGO detectors at Hanford, WA (H1) and Livingston, LA (L1). (**b**): The instrument noise for each detector near the time of the signal detection reprinted from [179].

In 2015, LIGO made the first direct detection of gravitational waves, reported in 2016 [179], as shown in Figure 19. This confirmed a key prediction of general relativity and opened a new era of observational astrophysics. Later detections, including those from neutron star pairs, have expanded our understanding of how compact objects form and evolve [180,181]. These discoveries have also encouraged new research across fields like gravitational physics, high-energy astrophysics, and cosmology. After the first detections, LIGO improved its performance by increasing laser power, enhancing mirror coatings, reducing seismic noise, and using quantum noise reduction methods such as squeezed light [182,183,184]. Current and planned upgrades aim for higher sensitivity, enabling more frequent observations and the possibility of detecting signals that were previously too weak to observe. Future third-generation detectors, like the Einstein Telescope and Cosmic Explorer, may reveal even fainter sources and deepen our understanding of fundamental physics.

By pushing the boundaries of precision measurement, LIGO confirms basic principles of gravity and helps shape our view of the universe. Its work also shows how optical interferometry can advance metrology and enhance our ability to measure length at an unprecedented level of detail.

### 3.10. Optical Coherence Tomography

Optical Coherence Tomography (OCT) technology was first proposed in 1991 by David Huang. Based on the principle of low-coherence light interference, OCT offers high-resolution, high signal-to-noise ratio (SNR), noninvasive, nondestructive, and three-dimensional imaging capabilities. It enables tomographic imaging of the internal microstructures of biological tissues, showing broad application prospects in fields such as biomedicine and materials science. Therefore, it is vividly referred to as “optical biopsy” [185]. Over nearly 30 years of development, OCT has evolved from early time-domain OCT (TD-OCT) [186,187,188] to frequency-domain OCT (FD-OCT) [189], and more recently to swept-source OCT (SS-OCT) [190], achieving significant breakthroughs in imaging resolution, SNR, and imaging speed [191].

**Figure 20:**
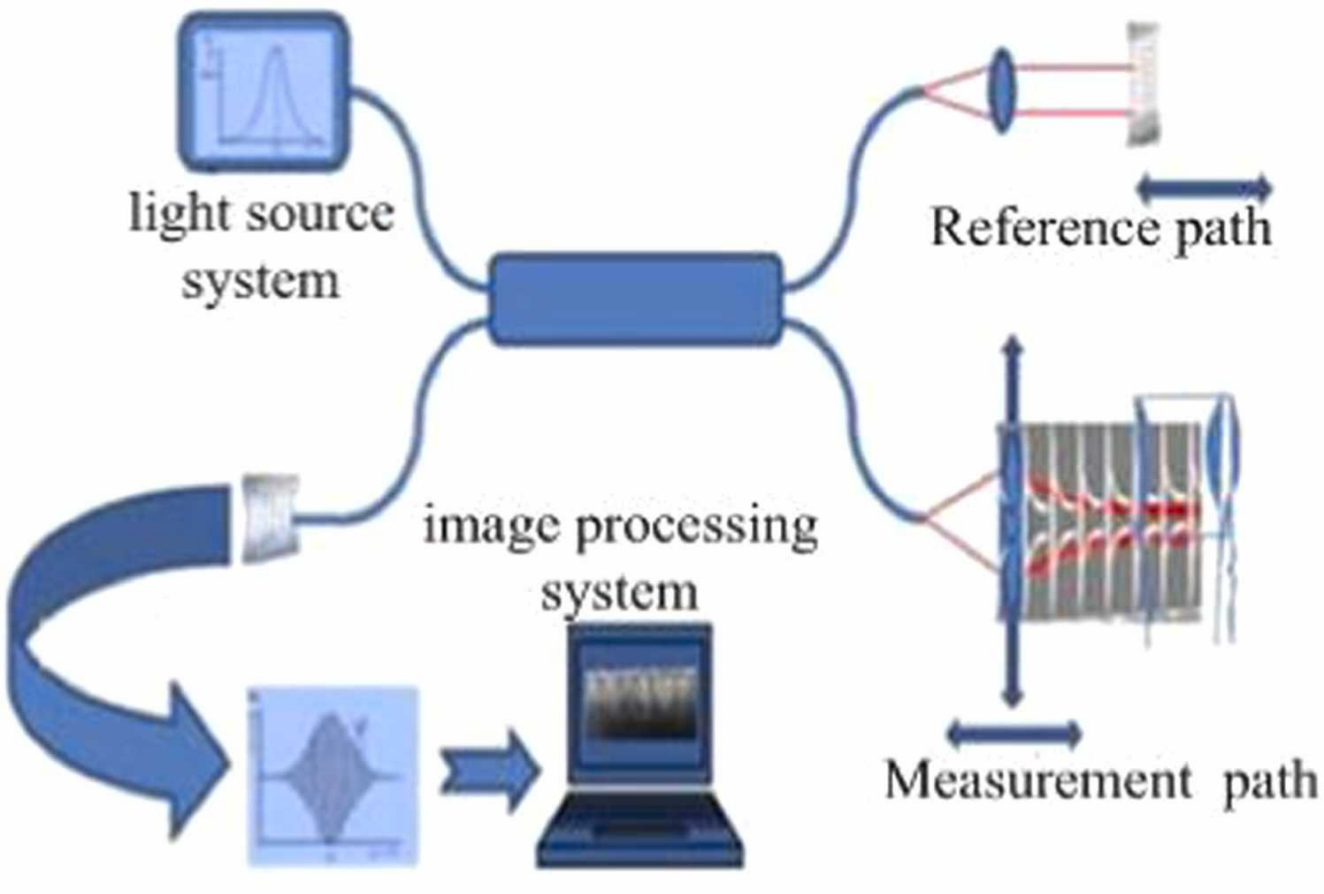
Schematic diagram of OCT measurement system, reprinted from [198], copyright 2022 Elsevier.

The basic principle of frequency-domain OCT (FD-OCT) is shown in Figure 20. The system consists of a low-coherence broadband light source, a sample arm, and a reference arm. Low-coherence light is split by a fiber coupler into two beams that enter the sample and reference arms, respectively. The back-scattered light from the sample arm interferes with the reference light from the reference arm in the fiber coupler. The interference signal is obtained through spectral separation and then reconstructed into axial depth information using Fourier transform, completing data acquisition and processing. Compared to time-domain OCT, FD-OCT uses a spectrometer composed of array detectors and gratings, significantly reducing scanning time while improving system detection sensitivity and SNR [192,193,194,195,196,197].

In 1991, David Huang proposed and used time-domain OCT technology for the detection and imaging of the human retina and coronary arteries [199]. In 1995, A. F. Ferche’s team introduced the concept of frequency-domain OCT [189], replacing the photodetector with a spectrometer, officially transitioning time-domain OCT to frequency-domain OCT, greatly reducing imaging time. In 1997, S. R. Chinn et al. from MIT [200] formally introduced swept-source OCT, using a swept laser source with a scanning rate of up to 10 Hz to replace the broadband light source. They successfully performed tomographic imaging of a 150 µm thick glass cover slip, obtaining relatively clear longitudinal imaging results. In 2020, Wartak et al. [201] used an FD-OCT system to achieve corneal microstructure imaging with 1 µm axial resolution and 1.5 µm lateral resolution. In the same year, Auksorius et al. [202] used FD-OCT (Fourier Domain Optical Coherence Tomography) for non-contact in vivo corneal volumetric imaging, acquiring data from a 615 µm × 615 µm field of view in just 8.6 ms.

## 4. Optical Frequency Comb Interferometry

**Figure 21:**
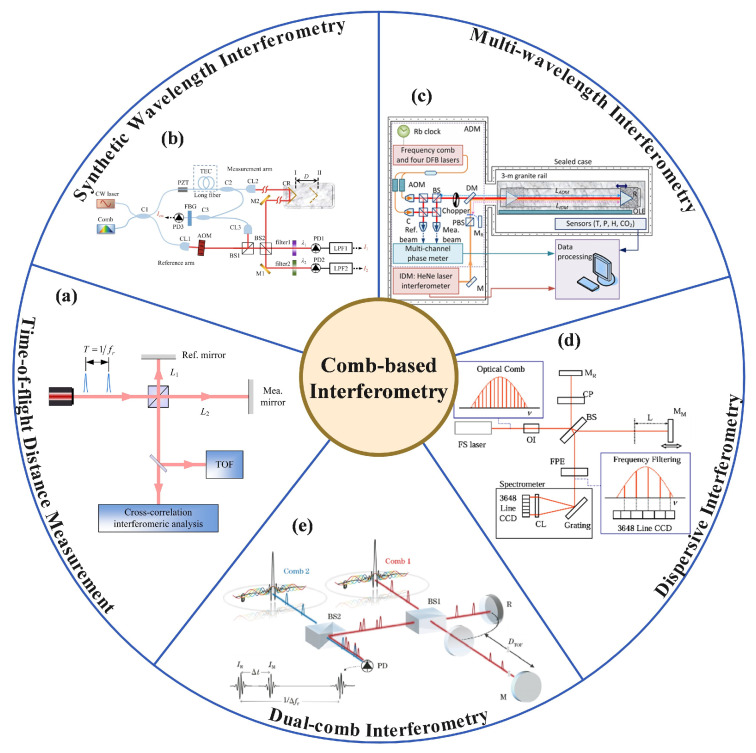
Key techniques in optical frequency comb: (**a**) The TOF length measurement method; (**b**) Schematic diagram of a synthetic wavelength interferometry, reprinted from [204]; (**c**) Schematic diagram of a multi-wavelength absolute length measurement interferometry, reprinted from [205]; (**d**) Schematic diagram of a sispersive interferometry using femtosecond laser pulses, reprinted from [206]; (**e**) Schematic diagram of dual-comb ranging system, reprinted from [73].

The optical frequency comb is a new type of light source with a comb-like spectral structure, representing a milestone breakthrough in laser technology. Compared to ordinary ultrashort pulse lasers, the optical frequency comb not only precisely links optical frequency standards with microwave frequency standards but also offers high stability, narrow pulse width, and a broad spectral range. It provides a stable time-frequency reference, ensuring high-precision measurements and offering a new technological approach for high-precision absolute length measurement. In the frequency domain, an optical frequency comb presents hundreds of thousands to millions of discrete, equally spaced spectral lines, while in the time domain, it appears as an ultrashort pulse signal with a pulse width at the femtosecond level. The frequency $f_{N}$ of the *N*th comb tooth is given by the equation:

$$f_{N}=N\cdot f_{\mathrm{rep}}+f_{\mathrm{ceo}}$$

where $f_{\mathrm{rep}}$ is the repetition frequency of the comb, and $f_{\mathrm{ceo}}$ is the carrier-envelope offset frequency. The locking of $f_{\mathrm{rep}}$ and $f_{\mathrm{ceo}}$ is typically achieved through cavity length adjustment, feedback control, and the “f-2f” self-referencing method [203]. After strict locking, the optical frequency comb has an extremely narrow linewidth, stable frequency, and phase, with each comb tooth representing a fixed wavelength, making it a precise ruler for measuring optical frequencies. The optical frequency comb has the ability to transmit the precision of atomic clocks to all wavelengths with wavelength uncertainty reaching the $10^{-18}$ level, with potential for further improvement. The precise connection between optical and microwave frequencies allows mature frequency synthesis techniques in the microwave domain to be applied to fine optical frequency control. Thanks to the unique phase-locking mechanism of the optical frequency comb, the phase difference between adjacent pulses remains fixed, meeting coherence conditions, which enables interference between different pulses and provides the necessary conditions for comb-based applications. Basic methods of comb-based interferometry are summarized as shown in Figure 21.

### 4.1. Time-of-Flight Method

The Time-Of-Flight (TOF) method determines distance by emitting a light pulse and receiving the signal reflected by the target. The pulse’s travel time through space is measured to calculate the distance. Traditional TOF methods measure the travel time of a laser over the distance, combined with the speed of light to obtain the distance result. However, its precision is limited by the timing circuits, typically achieving millimeter-level accuracy. By utilizing the time-domain characteristics of the optical frequency comb, the method of acquiring flight time can be improved, thereby enhancing length measurement accuracy.

By adjusting the repetition frequency of the optical frequency comb or the reference arm length, high-precision arbitrary length measurement can be achieved over a large range. This method was proposed in 2004 by Jun Ye [207]. As illustrated in Figure 21a, the pulse output of the optical frequency comb is split into reference and measurement beams by a beam splitter. The lengths of the reference arm ($L_{1}$) and measurement arm ($L_{2}$) represent the measured distance. This method requires a photodetector to capture the pulse intervals and an optical cross-correlator to detect interference fringes. When the repetition frequency is adjusted, the pulse interval changes. By measuring the pulse interval before and after adjustment and the repetition frequency, a rough estimate of the measured distance *D* can be obtained. Then, by fine-tuning the repetition frequency so that the two pulses overlap, interference fringes are produced, yielding a precise measurement of *D*, achieving sub-wavelength-level accuracy. It is important to note that for short distances, where the repetition frequency adjustment range is limited, pulse overlap cannot be achieved, resulting in millimeter-level measurement accuracy. M. Cui from Delft University of Technology experimentally validated this method, analyzed and corrected errors, and achieved a measurement accuracy of 2 µm over a distance of 50 m [208,209].

**Figure 22:**
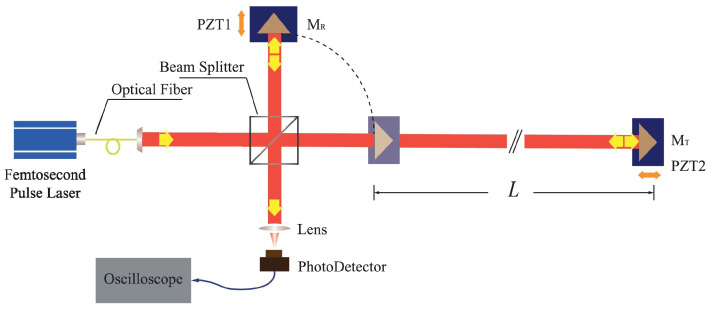
Schematic of TOF method, reprinted with permission from [212], copyright 2018 Elsevier.

In 2010, Joohyung Lee from the Korea Advanced Institute of Science and Technology (KAIST) used a balanced optical cross-correlation method to detect pulse delays. By utilizing a nonlinear crystal to convert time-domain correlation information into optical cross-correlation intensity, long-distance measurements of up to 700 m were achieved, with Allan variance reduced to 7 nm for an averaging time of 1 s [210]. In 2011, Dong Wei proposed a TOF method based on multi-pulse sequence interference of a femtosecond optical frequency comb, using a reference arm scanning method. The experimental results demonstrated a measurement uncertainty of $10^{-8}$ and a distance resolution of 4 nm [211]. In 2018, Yang Liu [212] from Tianjin University proposed an optimization scheme for pulse alignment and introduced an averaging method based on the symmetry of the time-domain cross-correlation function, improving the standard deviation of distance measurement results, as shown in Figure 22. In 2021, Jihui Zheng from Tianjin University used a silicon nitride micro-ring soliton optical frequency comb as a light source for distance measurement experiments. At a measured distance of 1.5 m, the accuracy was better than 384 nm, with a standard deviation of less than 137 nm [213].

### 4.2. Synthetic Wavelength Interferometry

The synthetic wavelength interferometry measures distance information by observing the phase variation of the beat signals between the comb teeth of an optical frequency comb. In 2000, Minoshima K. first proposed this method, using a femtosecond optical frequency comb as the light source [214]. She measured the phase of the beat signals between different comb teeth and synthesized the distance measurement results. In the experiment, three different beat signals were selected for measurement, and a resolution of 50 µm was achieved over a 240 m distance range, with a measurement uncertainty of $8\times 10^{-6}$. Later in 2010, she used the 821st harmonic signal of the optical frequency comb’s repetition frequency for distance measurement, reducing the measurement standard deviation to 200 nm [215]. For large distances, variations in the refractive index of air can limit measurement accuracy. To address this issue, Minoshima’s team applied a dual-wavelength interferometry method to correct the air refractive index. By measuring distances simultaneously with fundamental and second harmonic frequencies, and calibrating based on the combined results, they achieved a distance measurement uncertainty of the order of $10^{-8}$ [216,217,218].

**Figure 23:**
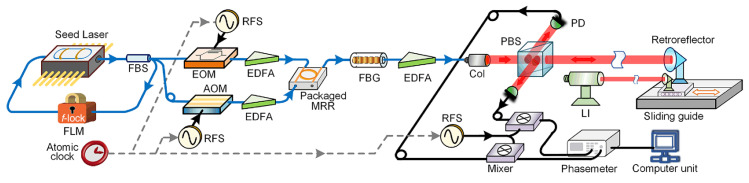
Schematic of soliton microcomb-based synthetic wavelength interferometry, reprinted from [222].

In 2010, Doloca R. N. from PTB built on the synthetic wavelength interferometry method, proposing a phase-based distance measurement method using comb teeth. They added a fixed-length measurement path to reduce the effects of temperature changes and wire stress, achieving a measurement accuracy of ±10 µm over a 100 m distance [219]. In 2014, Yoon-Soo Jang from KAIST extended the measurement range of the synthetic wavelength interferometry method by fine-tuning the repetition frequency of the optical frequency comb, achieving an absolute distance measurement of 13.3 m with an accuracy of 31.2 µm and a repeatability of 9.5 µm [220]. In 2018, Xianyu Zhao from Tianjin University proposed a three-comb synthetic wavelength interferometry method. They used three electro-optically modulated optical frequency combs with different repetition frequencies, employing 50 comb teeth near the central frequency for wavelength synthesis. The minimum synthetic wavelength’s measurement range was 0.3 mm, and within an 80 m measurement range, the distance measurement error was less than 750 nm [221]. Meanwhile, Guanhao Wu presented a synthetic-wavelength-based heterodyne interferometry of optical frequency combs for absolute distance measurement, achieving an accuracy of 75 nm in the 350 mm consecutive measurement range [204]. In 2023, Mingyue Yang developed a soliton micro-comb-based synthetic wavelength interferometry method, as illustrated in Figure 23. They stabilized the repetition frequency of the soliton micro-comb at 48.98 GHz ± 0.1 Hz using an injection locking method, achieving an Allan variance of 56.2 nm with an averaging time of 50 ms [222].

### 4.3. Multi-Wavelength Interferometry

The multi-wavelength interferometry method measures the distance by simultaneously using multiple different single-frequency laser beams, and the distance is calculated based on the phases of the different wavelengths. Since an optical frequency comb has numerous stable comb teeth, it is highly suitable as a light source or reference for multi-wavelength interferometry.

**Figure 24:**
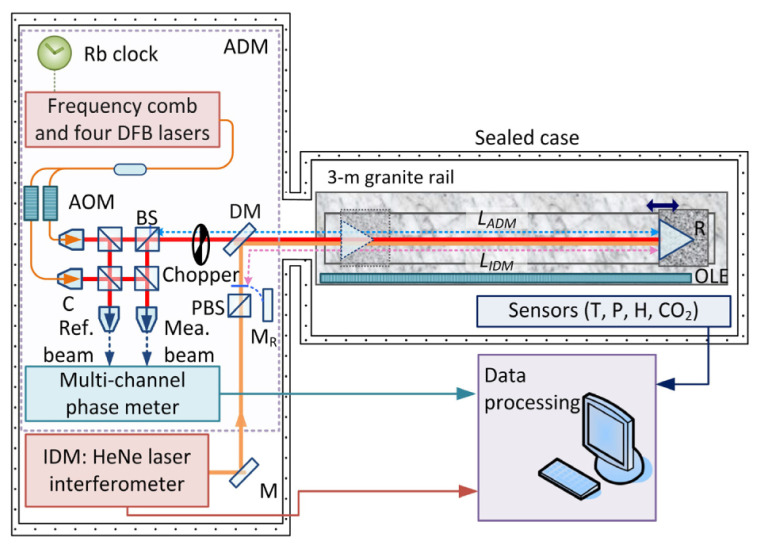
Multi-wavelength absolute length measurement interferometry, reprinted from [205].

In 2006, Jonghan Jin from KAIST proposed a multi-wavelength interferometry method based on an optical frequency comb. A continuous-wave laser was locked to the optical frequency comb, and the generated stable single-frequency light was used for interferometric measurement. The measurement distance was calculated by synthesizing the phase values of multiple wavelengths, achieving a measurement error of 15 nm [223]. That same year, Nicolas Schuhler locked two continuous-wave lasers with close wavelengths to different comb teeth of the same optical frequency comb, producing a synthetic wavelength of 90 µm with a relative uncertainty better than $2\times 10^{-7}$ [16]. In 2008, Yves Salvadé introduced a heterodyne detection mechanism based on the dual-wavelength interferometry method. This approach achieved a measurement accuracy of 8 nm over an 800 mm range when the target object moved at a speed of 50 mm/s [17]. In 2014, Guochao Wang conducted research on wavelength selection and unambiguous range in multi-wavelength interferometry, proposing a method for secondary synthetic wavelengths to extend the unambiguous range. Their research verified that a five-wavelength interferometry system could achieve an unambiguous range of several hundred millimeters [224]. In 2015, Guochao Wang, in collaboration with KAIST, conducted multi-wavelength interferometric distance measurement experiments using four lasers locked to an optical frequency comb, as illustrated in Figure 24. At a distance of 1 m, the linear error was 61.9 nm [205]. The different single-frequency lights are separated using the fiber Bragg grating (FBG). The phase values of each single-frequency light are detected, and the distance is calculated using the excess fraction method.

### 4.4. Dispersive Interferometry

The principle of dispersive interferometry is similar to that of white-light interferometry. Since an optical frequency comb has numerous comb teeth, these coherent longitudinal mode components can ensure spectral interference signals over a large range, greatly extending the measurement range. In 2006, Joo K. N. from KAIST proposed a dispersive interferometry method based on an optical frequency comb, achieving high-precision absolute distance measurement [206]. The measurement setup is shown in the Figure 21d. The femtosecond pulses generated by the optical frequency comb enter the interferometric optical path through an optical isolator. In the interferometric path, the reference mirror remains stationary, while the measurement mirror moves along the measurement axis. The measurement and reference beams are combined, filtered by a Fabry–Perot cavity, and then directed into a spectrometer. The spectrometer, composed of a grating and a linear CCD array, diffracts the combined light onto the CCD, where the interference fringes are detected, and the distance is measured. This method achieved a resolution of 7 nm at a measurement distance of 0.89 m, with an unambiguous distance of 1.46 mm.

**Figure 25:**
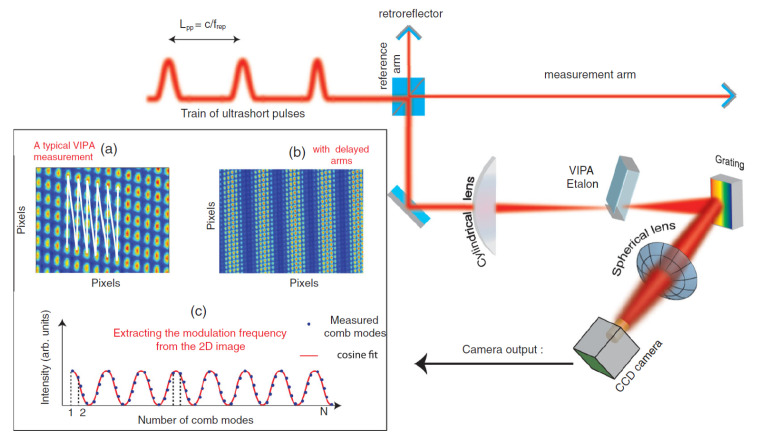
Schematic overview of the setup combining dispersive interferometry with multi-wavelength interferometry, reprinted with permission from [143], copyright 2012 APS.

In 2011, M. Cui employed dispersive interferometry to measure a distance of 50 m in air. By measuring small displacements instead of spectrometer calibration, a distance measurement accuracy of 1.5 µm was achieved [225]. In 2012, S. A. Van Den Berg conducted distance measurement experiments combining multi-wavelength interferometry and dispersive interferometry, and the setup is illustrated in Figure 25. Using a 1 GHz repetition-rate optical frequency comb as the light source, they measured the spectral phase delay introduced by each longitudinal mode at the measured distance, achieving a measurement accuracy of λ/30 with an unambiguous range of 15 cm [143]. In 2015, the same team measured a distance of 50 m in air using this method, achieving a measurement uncertainty of less than 1 µm [226]. That same year, Hanzhong Wu from Tianjin University designed a combined dispersive interferometry distance measurement system. Using an unbalanced Mach–Zehnder interferometric optical path, they eliminated the measurement dead zone. At a measurement distance of 1 m, the error was less than 1.5 µm, and at a distance of 75 m, the error was less than 25 µm, with a relative measurement accuracy of $3.3\times 10^{-7}$ [227].

**Figure 26:**
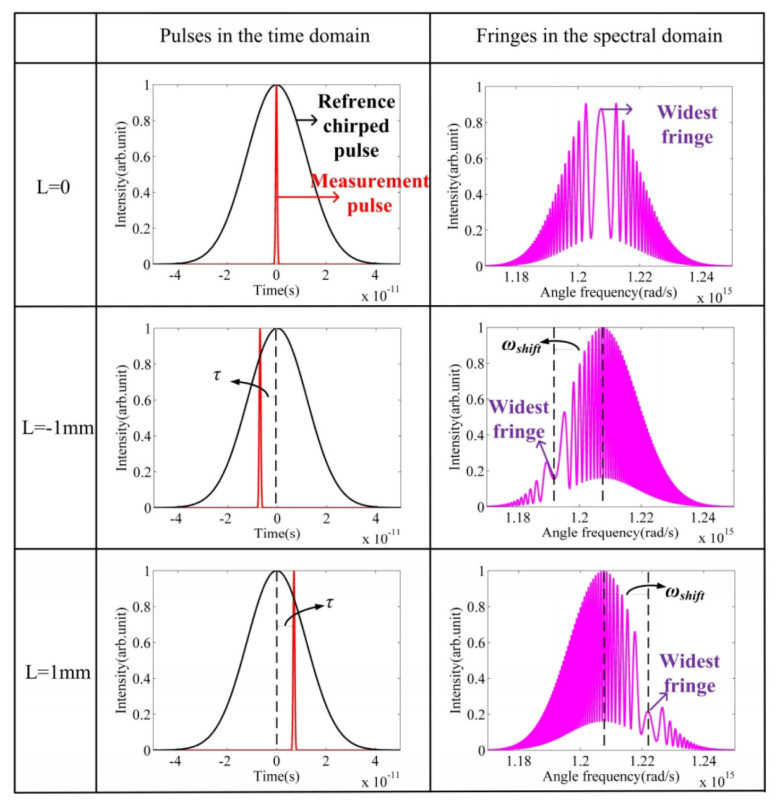
Simulations of chirped pulse interferometry with difference time delays, reprinted from [228].

It is worth mentioning that the fringe of the dispersive interferometry will be different if the pulse propagates through the strongly dispersive media, leading to the emergence of chirped pulse interferometry [228]. In 1994, Minoshima et al. first proposed the basic concept of using chirped femtosecond pulses for distance measurement [229]. They measured an area composing three flat steps made of three gauge blocks of different lengths with an accuracy of better than 0.3 mm. Using the powerful tool of an optical frequency comb, chirped pulse interferometry has been further developed. In 2015, Hanzhong Wu et al. proposed a method for absolute distance measurement by chirped pulse interferometry using a frequency comb, achieving an agreement within 26 µm in a range up to 65 m compared with a reference distance meter [228]. They conducted simulations of chirped pulse interferometry with difference time delays, and the fringes are shown in Figure 26. The widest fringe corresponds to a balanced wavelength that is related to the measurement distance. Later in 2019, they further conducted underwater experiments, and achieving an accuracy of 100 µm at 8 m range [230].

### 4.5. Dual-Comb Interferometry

**Figure 27:**
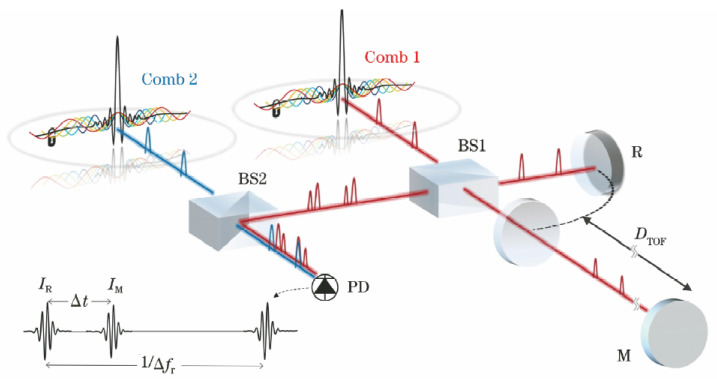
Schematic of dual-comb ranging system with orthogonal polarization structure, reprinted from [73].

Dual-comb interferometry employs two optical frequency combs with slightly different repetition frequencies as the light sources. The principle of length measurement using dual-comb interferometry is shown in Figure 27. The pulse sequence from comb 1 is divided into two paths by beam splitter 1 in the Michelson interferometry setup. After being reflected by the reference and measurement mirrors, the two pulse sequences, now delayed by a certain time, are recombined at beam splitter 1. These pulses are then combined with sampling pulses emitted by comb 2 at beam splitter 2, and the result is detected by the PD. This method was first proposed by Coddington I. [231] in 2009, who conducted distance measurement experiments using it. Within an unambiguous range of 1.5 m and with a response time of 200 µs, absolute distance measurements with an Allan variance of 3 µm were achieved. By further using the optical carrier phase from pulse interference, a measurement repeatability better than 5 nm was obtained with an averaging time of 60 ms.

In 2011, T. Liu [232] proposed an asynchronous optical sampling absolute distance measurement method using two free-running femtosecond fiber lasers in a dual-comb setup. This method does not require precise locking of the repetition frequency and carrier-envelope offset frequency of the local oscillator and probe combs, thereby reducing system complexity. With a measurement distance of 1 m and an averaging time of 0.8 ms, the measurement accuracy reached 1 µm; for an averaging time of 20 ms, the measurement accuracy improved to better than 200 nm. In 2013, J. Lee [233] achieved absolute distance measurement using the dual-comb method with balanced cross-correlation detection. By making the reference and measurement beams orthogonally polarized, the dead zones were eliminated. Additionally, the repetition frequency of the optical frequency comb was fine-tuned to extend the measurement range. At a distance of 69.3 m and an averaging time of 1 s, the measurement accuracy was approximately 170 µm.

That same year, Guochao Wang [234] proposed a large-scale multi-heterodyne dual-comb absolute distance measurement method. By using two optical frequency combs with differing repetition frequencies to perform multi-heterodyne interference, high-precision absolute distance measurements were theoretically achievable by combining single-line demodulation techniques, which were validated through numerical simulations. In 2015, Yuepeng Li [235] from Tianjin University built a large-scale absolute distance measurement system based on dual-comb interferometry. A comparison test conducted over a range exceeding 65 m showed that, with an averaging time of 0.5 s, the standard deviation was less than 6 µm.

In 2018, Zebin Zhu [236] from Tsinghua University proposed a stable-phase dual-comb synthetic wavelength absolute distance measurement system. This system balanced the large unambiguous range and high measurement accuracy through a synthetic wavelength chain. A distance measurement experiment over a range of 1.5 mm was conducted at a 1.5 m distance, achieving a repeatability of 3 nm with an averaging time of 10 ms. In 2019, this team applied dual-wavelength interferometry to compensate for air refractive index variations, achieving a distance measurement accuracy of 46 nm within a 2.7 m unambiguous range [237].

In 2020, Kim W. [238] from KAIST used the balanced cross-correlation method based on TOF to achieve multi-target distance measurement along the optical path. At a distance of 3 m and with a repetition frequency difference of 25 kHz, the standard uncertainty was measured at 0.986 µm with a 0.5 ms averaging time. In 2021, Mitchell T. [239] locked the repetition and carrier-envelope offset frequencies of two optical frequency combs with a repetition frequency difference of 130 kHz. Using the phase method for distance measurement, a measurement accuracy of 5 nm was achieved with an averaging time of 100 ms. In 2022, Ruilin Jiang [240] from Tsinghua University used a dual-channel optical filtering structure to avoid frequency aliasing. Under free-running conditions, the dual-comb distance measurement system operated stably for 60 min, achieving a repeatability of 6 µm.

## 5. Discussions

### 5.1. Error Analysis and Compensation Methods

**Figure 28:**
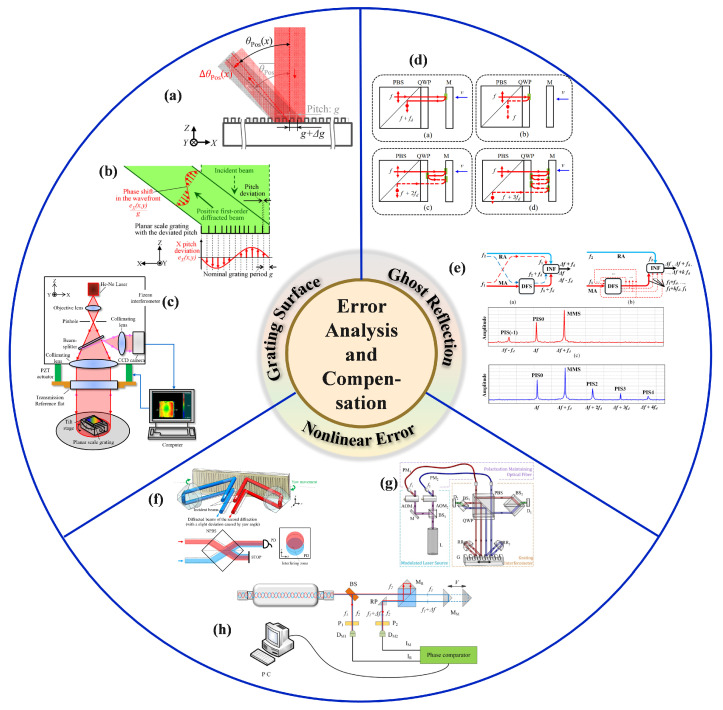
Error analysis and compensation methods. (**a**) Grating pitch error evaluation and analysis technology in 2021, reprinted with permission from [57], copyright 2021 Elsevier. (**b**) A rapid assessment method for grating pitch and waviness in 2010, reprinted with permission from [241], copyright 2010 Elsevier. (**c**) Uncertainty evaluation methods for plane gratings in 2018, reprinted from [242]. (**d**) Analysis and correction method of nonlinear error of ghost reflection in 2018, reprinted from [243]. (**e**) Nonlinear model evaluation of heterodyne interferometry in 2015, reprinted from [244]. (**f**) Double-diffraction grating spatial separation heterodyne grating interferometry in 2019, reprinted from [245]. (**g**) Spatially separated heterodyne grating interferometry in 2017, reprinted from [31]. (**h**) Nonlinear error analysis of dual-frequency interferometry in 2019, reprinted with permission from [246], copyright 2019 Elsevier.

This section delves into the innovative use of spatially separated structures as a solution. This approach adeptly circumvents the issues of polarization and frequency mixing, prevalent in common-path systems, thereby substantially reducing periodic nonlinearity errors. By exploring these advanced optical design methodologies, this section provides valuable insights into enhancing the accuracy and reliability of grating and laser interferometrys, catering to the escalating precision demands in various fields of metrology; the specific method is shown in Figure 28.

Recent advancements in grating fabrication technologies have played a pivotal role in enhancing the precision and uniformity of gratings used in multi-degree-of-freedom (DOF) measurement systems. In 2013, Li et al. [247] demonstrated the fabrication of scale gratings for surface encoders using laser interference lithography with 405 nm laser diodes, achieving high precision in grating pitch and surface smoothness. Building on this foundation, Li et al. [248] introduced an orthogonal two-axis Lloyd’s mirror system in 2018 for the holographic fabrication of large-area two-dimensional planar scale gratings, which significantly improved the scalability and uniformity of grating patterns. In 2021, Xue et al. [249] developed a polarized holographic lithography system capable of high-uniformity microscale patterning with periodic tunability, while Wang et al. [250] fabricated planar diffractive gratings for magneto-optical trap applications, further refining the precision and functional versatility of grating structures. Most recently, in 2023, Xue et al. [251] advanced the field by developing a dielectric-film-based polarization modulation scheme for patterning highly uniform two-dimensional array structures with periodic tunability, thereby enhancing the adaptability and precision of grating fabrication processes.

These technological improvements in grating manufacturing have a direct impact on the accuracy and reliability of precision measurement systems. Recent research has focused extensively on understanding the impact of optical component quality, particularly grating pitch accuracy, on measurement errors. Various representative works have addressed different aspects of this issue. For instance, in 2021, Quan et al. proposed a novel technology for evaluating and analyzing grating pitch errors, which provides insights into systematic deviations and error sources associated with grating manufacturing processes [57]. Earlier, Gao et al. (2010) introduced a rapid assessment method aimed at efficiently measuring grating pitch and surface waviness, enabling quicker quality control in practical applications [241]. Moreover, Xiong et al. (2018) developed uncertainty evaluation methods specifically designed for plane gratings, focusing on quantifying the measurement uncertainties that arise from imperfections in grating fabrication [242]. These studies collectively highlight the critical role of grating quality in precision measurements and have laid the groundwork for further advancements in this field. Additionally, Guan et al. [252] developed a differential interferometric heterodyne encoder that achieves a periodic nonlinearity of 30 picometers and sub-nanometer stability. The system offers enhanced precision and stability, making it suitable for applications demanding high-resolution displacement measurements. Collectively, these advancements in grating processing and error analysis underscore the continuous efforts to mitigate measurement inaccuracies, thereby enabling more robust and reliable multi-DOF precision measurement systems.

Addressing issues related to ghost reflections and periodic nonlinear errors in measurement systems has been a significant area of research, with various methods proposed to mitigate these challenges. Fu et al. (2018) presented an analysis and correction method specifically targeting the nonlinear errors caused by ghost reflections, providing a systematic approach to identify and compensate for such errors within optical systems [243]. In a related study, Hu et al. (2015) developed a nonlinear model evaluation technique for heterodyne interferometries, which enhanced the understanding of the root causes of periodic nonlinearities and offered a basis for more effective error mitigation strategies [244]. Building on these concepts, Chang et al. (2019) introduced a double-diffraction grating spatial separation heterodyne grating interferometry, a novel design aimed at minimizing the effects of periodic nonlinear errors by enhancing the separation between signal components [245]. Furthermore, Fu et al. (2020) proposed real-time compensation algorithms for heterodyne interferometries, enabling dynamic correction of nonlinear errors during measurements, thereby improving measurement accuracy and stability [253]. Yang et al. (2019) also contributed to this field by analyzing the nonlinear errors of dual-frequency interferometries, providing a deeper insight into error sources and correction mechanisms [246]. Wang et al. [254] proposed a design and parameter optimization method for zero-position codes, incorporating diffraction considerations and utilizing deep learning generative adversarial networks. This approach enhanced the accuracy of zero-positioning by optimizing diffraction patterns, demonstrating the potential of advanced AI techniques in precision engineering applications. Together, these studies represent a comprehensive effort to address and mitigate the impact of ghost reflections and periodic nonlinearities, contributing to more precise and reliable measurement systems.

### 5.2. Phase Measurement Methods

The process of displacement measurement through interferometry can usually be divided into four key parts: laser source, interference optical path, photodetector, and signal processing. The basic principle of measurement by laser or grating interferometry is the Doppler shift. That is, the movement of the measured target will cause a phase difference between the measurement signal and the reference signal. The core of the signal processing system is to accurately measure this phase difference through waveforms, which is crucial for accurate displacement calculation. The core of this section is an exploration of mainstream phase measurement methods, which are mainly divided into four main types: mixed frequency-based methods, time resolution-based methods, amplitude resolution-based methods, and hybrid methods [255,256,257].

**Figure 29:**
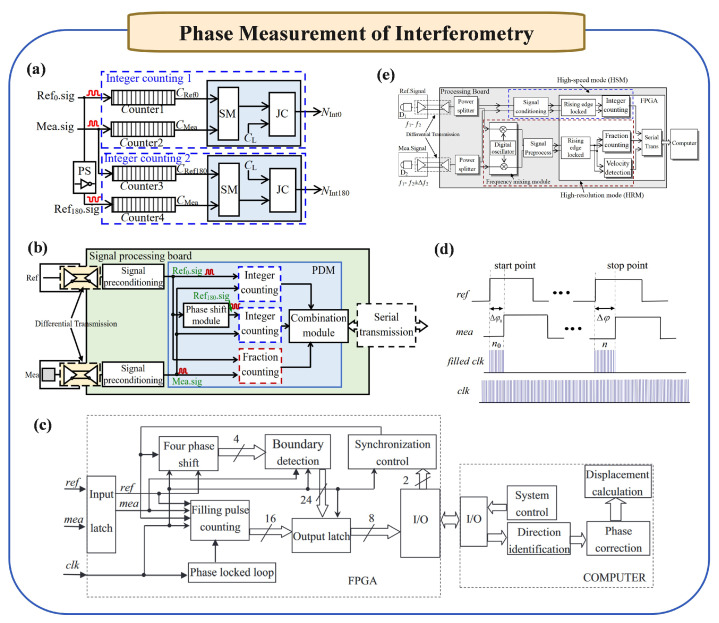
Phase measurement of Interferometry. (**a**,**b**) Phase-shift-based interferometric signal processing methods in 2018, reprinted from [256]. (**c**,**d**) Phase correction algorithm in heterodyne interferometry in 2014, reprinted with permission from [258], copyright 2014 Elsevier. (**e**) Heterodyne interferometry based on phase-locked signal processing algorithm in 2013, reprinted from [259].

Recently, Hu et al. (2023) proposed a high-precision phase resolution method based on a digital dual-frequency comb, which uses the advantages of dual-comb technology to achieve superior phase sensitivity and measurement precision [255]. Earlier, Zhang et al. (see Figure 29a,b) introduced phase-shift-based interferometric signal processing methods, which utilize controlled phase shifts to extract phase information more effectively, improving the robustness of the measurements against environmental fluctuations [256]. These advancements have paved the way for more precise phase determination, especially in applications where high resolution is essential.

In addition, researchers have focused on compensating for errors that affect phase measurements. For instance, Wang et al. (2016) addressed the issue of data age errors in non-constant velocity metrology, introducing a compensation method to correct for timing discrepancies that can lead to phase errors during dynamic measurements [260]. In the realm of heterodyne interferometry, Yang et al. (see Figure 29c,d) developed a phase-correction algorithm tailored for heterodyne interferometries, focused on improving the precision of phase measurement through real-time adjustments [258]. Zhang et al. (see Figure 29e) proposed a phase-locked signal processing algorithm to improve phase stability and accuracy by minimizing phase drift and noise [259]. Together, these efforts represent significant steps toward enhancing the accuracy and reliability of phase measurement in interferometric systems.

## 6. Conclusions and Perspectives

Optical interferometry-based length measurement technology has been widely used in scientific research and industrial fields due to its advantages such as non-contact, high resolution, and large dynamic measurement range. A review focused on basic methods of optical interferometry is presented in this paper. The fundamental principles and research status of each method are introduced. A comprehensive analysis is conducted on common problems in optical interferometry, such as the influence of nonlinear periodic error and phase measurement accuracy. As a classic method for length measurement, optical interferometry still has great potential for development and plays an irreplaceable role in measurement applications with increasing precision requirements. Overall, in the field of precision measurement, laser interferometry and grating interferometry are pivotal technologies that have undergone significant advancements. However, several key challenges and opportunities for future development remain.

### 6.1. Grating Interferometry: Future Directions

In parallel, grating interferometry offers unique advantages for precision measurement, but it too faces challenges that require innovative solutions. A primary area for future research is the development of large-area grating fabrication techniques. The ability to produce composite gratings over extended ranges will not only expand the measurement travel but also enable periodic error correction, ensuring long-range accuracy. This requires breakthroughs in both grating fabrication technology and the integration of error correction mechanisms.

The trend toward modular and integrated systems also applies to grating interferometry. The further development of collimation modules for diffracted beams, along with the refinement of anti-reflective and high-reflective coatings, is essential to meet the demands of highly integrated measurement systems. Such developments will enable more compact and robust grating interferometry setups.

Precision improvement remains a focal point in grating interferometry. Chain-based error analysis models must be constructed to trace and systematically eliminate sources of measurement error. By understanding the error propagation pathways, targeted solutions can be developed to enhance measurement accuracy further. Additionally, improving the tolerance capacity of grating interferometries will be key to expanding their applicability. Tolerance to environmental variations, such as thermal drift, mechanical vibration, and alignment errors, will dictate their suitability for broader industrial applications.

### 6.2. Laser Interferometry: Future Directions

Laser interferometry, as the cornerstone of precision metrology, relies heavily on the performance of its light source, which serves as both the signal origin and the measurement reference. Future work should focus on improving frequency stability, as it directly impacts the accuracy of measurements. While current frequency stabilization techniques have achieved impressive results, novel stabilization methods must be explored to further enhance long-term stability, particularly in the context of sub-nanometer precision. Moreover, multi-axis integration in laser interferometries requires further investigation to meet the demands of multi-degree-of-freedom measurement tasks. The development of compact, integrated modules capable of handling multiple axes simultaneously is crucial for real-world applications, such as in advanced manufacturing and semiconductor processes.

Another critical area for future research is the adaptation of laser interferometry for vacuum environments. High-end applications like lithography systems demand novel structural designs that can maintain performance under vacuum conditions. This poses significant challenges in terms of optical component design, thermal management, and alignment precision. Additionally, with the increasing prevalence of high-speed measurement scenarios, the calibration and mitigation of dynamic errors become essential. Future studies should focus on developing dynamic error compensation techniques that can operate in real-time to correct for distortions introduced by rapid motion, ensuring accurate measurements in ever-faster systems.

### 6.3. Outlook

In conclusion, both laser interferometry and grating interferometry are expected to play increasingly important roles in the future of precision measurement. By addressing the challenges of frequency stabilization, multi-axis integration, vacuum environment adaptation, and dynamic error correction in laser interferometry, and by advancing large-area grating fabrication, integration, precision improvement, and tolerance enhancement in grating interferometry, we can unlock new levels of performance in precision measurement. These developments will not only push the boundaries of current technologies but also open the door to new applications in advanced manufacturing, semiconductor processing, and beyond.

## Figures and Tables

**Table 1:** Comparison of laser interferometries.

Method	Advantages	Challenges
Homodyne Interferometry	Simple setup; Compact structure; Low cost; High accuracy and sensitivity.	Sensitive to ambient light and environment; Combined light intensity is not stable; High requirement for environment.
Heterodyne Interferometry	High signal-to-noise ratio; Strong anti-interference ability to the environment; High accuracy; Large dynamic range	Target speed is limited; periodic nonlinear error needs to be further reduced.
Fabry–Perot Interferometry	High sensitivity; miniaturization and light weight; Multiplexing ability; Low requirements on measurement environment.	The relative accuracy of the measurement is limited by the cavity length; Folding structure or optical fiber structure is required to extend the measurement range.
Self-mixing Interferometry	Simple and compact structure; Low cost; Easy to collimate; Not affected by light source coherence or laser type.	The system debugging is complicated; The measurement accuracy is affected by the laser modulation performance
Multi-wavelength Interferometry	Fast and accurate absolute length measurement ability; No measurement dead area; Retain the resolution and accuracy of single wavelength interferometry over a large scale.	High requirements for laser frequency stability and synchronous phase measurement accuracy; Reasonable selection of wavelength combinations to build a synthetic wavelength chain; Integrated high-performance multi-wavelength light source is required.
Frequency-sweeping Interferometry	No measurement dead zone; No need for cooperative targets; High signal-to-noise ratio; Capable of large-scale absolute length measurement	Susceptible to laser mode hopping, frequency modulation nonlinearity, and environmental vibration; Signal spectrum broadening caused by fiber dispersion mismatch; FFT transform spectrum leakage.
White Light Interferometry	No phase ambiguity; Applicable to a wide range of materials and surface types; Insensitive to environmental fluctuations.	Interference fringe clarity is affected by coherence length; Lateral crosstalk; Data processing is computationally intensive; Real-time performance is affected by scanning rate.
